# Molecular mechanisms underlying heat or tetracycline treatments for citrus HLB control

**DOI:** 10.1038/s41438-018-0038-x

**Published:** 2018-06-01

**Authors:** Fang Ding, Victoria Allen, Weiqi Luo, Shouan Zhang, Yongping Duan

**Affiliations:** 1 Hubei Key Laboratory of PLant Pathology, Huazhong Agricultural University, 430070 Wuhan, Hubei P.R. China; 2 USDA-ARS-USHRL, Fort Pierce, FL 34945 USA; 3 Center for Integrated Pest Management, North Carolina State University, Raleigh, NC 27606 USA; 4 IFAS-TREC, University of Florida, Homestead, FL 33031 USA

## Abstract

Huanglongbing (HLB), a destructive plant bacterial disease, severely impedes worldwide citrus production. In our previous reports, we revealed the molecular mechanisms of host plant responses that underlie thermotherapy against HLB. In this study, we investigated the molecular mechanism underlying heat or tetracycline treatments on the HLB bacterium, ‘*Candidatus* Liberibacter asiaticus’ (Las) by focusing on Las prophage/phage conversion under stress conditions. By comparing the prophage FP1 and FP2 copy number to the copy number of 16S rDNA in HLB-affected plants, we found that the relative copy number of both FP1 and FP2 increased significantly, ranging from 3.4- to 6.7-fold change when Las-infected samples underwent a temperature shift from 23 to 37, 42 or 45 °C. When treated with tetracycline at 50–150 and 200–250 µg/ml, respectively, the relative copy number of both FP1 and FP2 increased by 3.4- to 6.0-fold. In addition, analyses of Las prophage structural gene and antirepressor gene copy numbers showed similar trends for all treatments. Furthermore, transmission electron microscopy provided direct evidence of lysogenic to lytic conversion upon temperature increase. These results not only provide new insight into the molecular mechanisms underlying heat or tetracycline treatment but also suggest a novel HLB control strategy by enhancing the endogenous conversion from Las prophages to phages.

## Citrus disease: Fighting one infection with another

Heat treatment to cure citrus plants of Huanglongbing (HLB) works not by killing the HLB bacteria directly but by triggering a virus to attack them. HLB, also known as citrus greening because it causes plants to produce green, bitter fruit, threatens citrus production worldwide. No commercial-scale treatments are available, but thermotherapy, growing plants at 40 °C or higher, has recently been shown to reduce symptoms. YongPing Duan and Fang Ding at the USDA Horticultural Research Laboratory in Florida investigated how the thermotherapy works. They found that heat treatment triggered a stress response in a virus asymptomatically infecting the bacteria, causing it to multiply rapidly and killing the HLB bacteria. Heat treatment eliminated both the bacteria and HLB symptoms; application of the antibiotic tetracycline produced the same effect. These results may help in identifying potential treatments for HLB.

## Introduction

Citrus Huanglongbing (HLB), also known as citrus greening, is one of the most destructive diseases that affects the citrus industry throughout the world. The causal agents are insect-vectored, phloem-limited, and fastidious α-proteobacteria, including ‘*Candidatus* Liberibacter asiaticus’ (Las), ‘*Ca*. L. africanus’ (Laf), and ‘*Ca*. L. americanus’ (Lam)^1–3^. Asian citrus psyllid, *Diaphorina citri* Kuwayama, *Cacopsylla* (*Psylla*) *citrisuga* (Hemiptera: Psyllidae)^4^, and African citrus psyllid, *Trioza erytreae* (Del Guercio), are the main transmission vectors of HLB pathogen^2^. All known citrus species and cultivars regardless of rootstock or scion are susceptible to HLB^5–8^, although a recent survey of 30 different genotypes of Florida citrus varieties showed different sensitivities to HLB bacteria^9^. The disease often displays diverse symptoms of yellow shoots, blotchy mottle on leaves, vein corking, die-back, and malformed fruit along with off flavored tastes^10,11^. Because of the systemic distribution of Las in infected plants, HLB is extremely difficult to cure. Although there has been limited success in treating individual trees for HLB^5,12,13^, no chemicals or protocols viable on a commercial scale are currently known. HLB continues to cause serious economic losses and has resulted in the destruction of tens of millions of citrus trees worldwide^2^.

Las was first detected in South Florida in August, 2005^1^ and is now widespread throughout the state with an estimated disease incidence of >95%^14^. HLB is now found in at least seven states in the United States, including Louisiana, Texas, and California, and threatens to destroy the entire U.S. citrus industry^11^. Currently, the most effective way to reduce economic loss from the disease is by controlling the citrus psyllid population^15^ and removing infected citrus trees. Recently, aggressive plant nutrition programs have been undertaken in order to increase production from infected trees^16^. In addition, heat treatment^17,18^, antimicrobial compounds^19^, injecting citrus trunks with bactericide^20^, and application of plant-defense inducers^21^ have shown the potential to mitigate HLB disease.

Bacteriophages play very important roles in community composition, evolution of their bacterial hosts, and the emergence of new pathogens^22–25^. Many phages possess a genetic switch that allows them to alternate between the lytic and lysogenic life cycles. Stresses such as sudden temperature increase, antibiotic compounds, and ultraviolet (UV) light can cause infected bacteria to enter a lytic cycle, wherein the prophage is excised and no longer lysogenic^26^. Conversion of phages may offer a selective advantage, as it allows gene transfer from host to host and dramatically affects host phenotype^27^. Bacteriophage-derived regions are responsible for chromosomal rearrangements and deletions, thus playing a decisive role in bacterial genomic diversity^28,29^. Increasing attention has been devoted to studying the biological and molecular characteristics of these particles. According to NCBI, about 500 phage genomes have been sequenced and deposited in GenBank (https://www.ncbi.nlm.nih.gov/). Recently published works demonstrate that at least two prophages (phages) are associated with Las^30,31^, named SC1/FP1 and SC2/FP2. SC1/FP1 encodes a putative holin and endolysin^32^, indicating the potential for lysogenic to lytic conversion. As previously noted, bacteriophages have been speculated to play a central role in their hosts. However, due to the present inability to culture HLB bacteria in vitro, the lysogenic conversion of HLB bacteriophages under stress conditions remains poorly understood. Although a recent study has shown that a small Wolbachia protein may act as a repressor of Las prophages’ lytic cycle in the psyllid^33^, factors that affect lysogenic to lytic conversion of Las prophages in citrus have not yet been identified.

Our previous study showed that controlled heat treatment dramatically reduced Las titer in both citrus and periwinkle plants infected with HLB bacterium, with Las becoming undetectable months after heat treatment^18^. A follow-up proteomics study demonstrated that heat treatment causes several chaperone-related proteins to be upregulated in citrus trees^34^. More recently, we analyzed the transcriptome of heat treated trees and showed that in HLB-affected citrus, posttreatment gene expression more closely resembled that of healthy controls^14^. Although these studies have helped elucidate how host plant physiology is altered by heat treatment, it remains unknown how Las physiology is altered by heat treatment. In addition to the positive host response induced by heat treatment and the direct antibiotic killing effect of tetracycline, understanding the mechanism by which Las bacteria are also eliminated via self-regulation of prophage/phage conversion triggered by stress conditions may be crucial to developing new HLB therapies.

We hypothesized that lysogenic conversion of HLB prophages may be involved in this process. In this study, we characterized copy number of specific FP1 and FP2 genes by real-time PCR. The goals of our research efforts were (1) to evaluate the effects of sudden temperature increases on prophages in HLB-affected citrus and periwinkle, (2) to investigate the effects of antibiotic stress (tetracycline) on prophage conversion in infected citrus samples, and (3) to compare the prophages’ responses to different stress conditions. Here we describe the responses of FP1 and FP2 to both temperature and antibiotic stress conditions. The results of this study both advance our understanding of the process of HLB bacteriophage conversion between lytic and lysogenic cycles and suggest a possible mechanism for the effectiveness of thermotherapy and antibiotics against Las.

## Materials and methods

### Plant material and growth conditions

Grapefruit (*Citrus paradisi*) and periwinkle (*Catharanthus roseus*, Pacifica XP White Vinca, Harris Seeds, New York) plants were inoculated by grafting as described previously^35^ using Las-infected citrus and periwinkle, respectively, and transmission was confirmed by quantitative PCR (qPCR). These infected plants were maintained in the insect-proof greenhouse at the USHRL facility in Ft. Pierce, FL. Temperature stress experiments were carried out in a CMP5000 growth chamber (Conviron Corp., Canada) with fluorescent lamps at 40% intensity, a 12 h/photoperiod, and 85% relative humidity unless otherwise stated. Before and after designated treatments, each plant was returned to the HLB-isolation greenhouse where they received weekly water and fertilizer management (Peters 20-10-20 Special).

### Heat stress treatment

One-year-old infected periwinkle or 2-year-old infected grapefruit (*Citrus paradisi*) (approximately 0.5–0.8 m in height) were moved from the greenhouse (35 °C) to the isolation room (23 °C) and maintained for 2 days before heat stress treatment. Plants were placed in a growth chamber programmed to 37 or 42 °C for 10 days. For each treatment, 3 leaf samples were picked from each plant at 2, 4, 6, 8, and 10 h, as well as at 1, 2, 3, 5, and 10 days after the temperature shift. For 45 °C treatment, samples were collected in the same way except that the last time point sampling was finished on day 3. Healthy control plants, including Las-negative periwinkle and Las-negative grapefruit, underwent the same heat treatments without adverse effects. Las-infected periwinkle and grapefruit without heat treatment kept in the greenhouse also were used as controls. Each experiment was conducted in triplicate with four plants. Before and after treatment, all plants were tested for ‘*Ca*. Liberibacter asiaticus’ using qPCR as described below.

### Antibiotic stress treatment

Experiments were conducted with symptomatic HLB-affected lemon (*Citrus limon*) branches about 0.5–0.8 cm in diameter. Each branch included approximately 10–12 leaves (Figure S1). The branches were dipped into 15 ml tubes (covered with aluminum foil paper) with different concentrations of oxytetracycline hydrochloride (#75966, Sigma) at 50, 100, 150, 200, and 250 µg/ml. HLB-affected lemon branches placed into water were used as a control. Leaf samples were collected at 1, 3, 5, 7, and 9 h post-antibiotic treatment and at 1, 2, and 3 days post-antibiotic treatment. Each experiment was conducted in triplicate with four branches.

### Transmission electron microscopy (TEM) of Las phages

Periwinkle leaves were collected at different time intervals after temperature shift from 23 to 45 °C. HLB-affected periwinkle leaves that remained at 23 °C were used as control. Leaf midrib samples were dissected and placed in a primary fixative of 2.5% glutaraldehyde and 2.5% formaldehyde in 0.1 M Cacodylate buffer (pH 7.2). After repeated rinsing in 0.1 M Cacodylate buffer, samples were fixed in 2% osmium tetroxide in 0.1 M Cacodylate buffer at 4 °C overnight; after rinsing, the samples were treated with 1% aqueous uranyl acetate at room temperature. Samples were slowly dehydrated in a graduated ethanol series to 100% ethanol and then 100% acetone. They were slowly and progressively infiltrated with a modified Spurr’s epoxy resin and polymerized at 60 °C. The leaf samples were briefly exposed to microwave radiation (model 34700, Pelco Biowave) at each step to aid penetration and infiltration. Polymerized samples were sectioned on a Reichert Ultracut R ultramicrotome, collected on formvar-coated copper slot grids, and viewed on a Hitachi H7000 transmission electron microscope at 100 Kv equipped with a Soft Imaging Systems (SIS) Valeta digital camera.

### Sample preparation and DNA extraction

For every plant, three symptomatic leaf samples were collected to determine the initial cycle threshold (Ct) values of Las before treatment and then collected again after treatment at the time points previously stated. In order to minimize the effects of Las’ uneven distribution, one leaf at the top and one leaf at the bottom of the branch were collected. Leaf tissue was immediately processed after collection or maintained at −20 °C in a freezer. Total genomic DNA was extracted using the Qiagen^®^ DNeasy Plant Mini Kit protocol with slight modification (Qiagen, Germantown, MD). Briefly, about 200 mg of leaf midrib tissue was chopped into tiny fragments and placed in sterilized 2 ml tubes with silicone-carbide shards and chrome-steel beads (2.3 mm in diameter), with 1.0 ml of AP1 extraction buffer. Tissue was then homogenized twice using a Fast Prep^®^-24 homogenizer (MP Biomedical, Solon, OH) at speed of 6.5 for 60 s. In all, 4 µl of RNase A was added before samples were incubated at 65 °C for 30 min. In all, 200 µl of AP2 buffer was added; samples were incubated on ice for 5 min and then centrifuged at 15,000 rpm for 5 min, after which the supernatant was transferred to the QIA shredder Mini spin column and centrifuged again at 15,000 rpm for 3 min. Flow through was transferred to a new 2 ml sterilized tube and 1.5 volumes of Buffer AP3 was added. A total of 650 µl of each sample was transferred to a DNeasy Mini spin column placed in a 2 ml collection tube and centrifuged at 8000 rpm for 1 min, and then the flow through was discarded. This was repeated until all of the sample had been processed. Columns were washed twice with wash buffer; DNA samples were eluted in 100 µl of sterilized water and stored at −20 °C for further analysis.

### Real-time PCR

Real-time PCR amplifications were performed in an Eppendorf Mastercycler^®^ realplex thermal cycler. The primers HLBasf and HLBr^36^ that specifically target the 16S rDNA region of ‘*Ca*. L. asiaticus’ were used as a reference for Las titer, and an additional primer set amplifying the putative Las origin of replication (*ori* C) was designed to confirm the Las response under different stress conditions. All primer sets targeting different prophage regions are listed in Table 1. Primers were designed using IDT SciTools PrimerQuest^SM^; a BLASTN search was performed to confirm the specificity of the primers designed. In order to obtain a maximum efficiency for each amplification reaction, all primers were 17–20 nucleotides long with melting temperatures of 60 °C. Dimer formation, heterodimer formation, and hairpin formation between primers and probes were all tested by the IDT SciTools PrimerQuest^SM^ software. All oligonucleotides were synthesized by Integrated DNA Technologies, Inc. (Coralville, IA).

**Table 1:** Oligonucleotide sequences of real-time PCR primers and probes used in this study

Name	Sequence (5’–3’)	Target gene or flanking region	Reference
Prophage FP1-specific primer			
FP1-gp110F	GAAGTGAGACGCCAGGAAAG	Possible holin	^30^
FP1-gp110R	TCGTACATGCACCCCTGATA		
FP1-gp235F	AAGGGGTCTAATCTACCGCC	Putative phage-related protein	^30^
FP1-gp235R	TTTGTATGGCTTGCCTCAAC		
Prophage FP2-specific primer			
FP2-gp240F	GCACGCATGGAGAGAGATTT	Putative trimeric autotransporter adhesin	^30^
FP2-gp240R	CCAGTCAAACCCTTTAGCCA		
FP2-gp065F	AATGTTTCCAAATCCGCAAG	Possible integrase	^30^
FP2-gp065R	GTATCGCAAGCTCAAGCACA		
Primers for both FP1 and FP2			
gp-025F	TATGAGGAGCTCTGGGGCTA	Putative phage tail fiber protein; lectin-binding	This study
gp-025R	TGATGTCGATGGGTTAAACG		
gp-030F	CAAACTCCCGTTTCACACCT	Putative phage structural protein	This study
gp-030R	CGCTTTCCCGTTCTGAAATA		
gp-035F	TCAAAGATGCGGTAATGCTG	Possible endolysin	This study
gp-035R	GGGCAGTGTTGAATGTTCCT		
gp-200F	CCTCTTCCGATAACGACGAA	Putative Bro-N family phage antirepressor	This study
gp-200R	AGCCACCTGAGACCTTGCTA		
Pro-R.O. F	CGCACGAGGTGTAGCTTATG	rep_origin in UF506	This study
Pro-R.O. R	GAGGACAGCCGACGATTACT		
Primers for Las			
Las-R.O. F	TTACCAATGCGGATGGTTCT	Las oriC region in psy62	This study
Las-R.O. R	ATCCGAATCCCTGTTGTGAT		
HLBasf^a^	TCGAGCGCGTATGC**G**AATACG	16S rDNA	^31^
HLBr	GCGTTATCCCGTAGAAAAAGGTAG		
HLBp	^b^AGACGGGTGAGTAACGCG^c^		

^a^Additional guanine nucleotide (bold, underlined “G”) was added to HLBas primer sequence^63^ based on 16S rDNA sequence in ‘*Ca*. Liberibacter asiaticus’ Psy62 genome and named as HLBasf

^b^6-FAM™ at 5’-end

^c^Iowa Black FQ at 3’-end

For SYBR real-time PCR, each reaction contained the following components at a total volume of 15 μl: 7.5 μl of SYBR® Green PCR Master Mix system (PERFECTA SYBR FASTMX LRX, VWR), 250 nM each of forward and reverse primer, and 1.0 µl of DNA template. The cycling protocol was as follows: 95 °C for 5 min, followed by 40 cycles of 30 s at 95 °C, then 60 °C for 30 s. For TaqMan real-time PCR, the reaction mixture was performed in a total volume of 15 µl: 7.5 µl of TaqMan PCR master mix (Applied Biosystems), 250 nM each primer, and 100 μM probe, with cycling conditions as in ref.^36^. All reactions were done in duplicate or triplicate using fast and TSP-heated lid temperature mode.

### Amplification efficiency for real-time PCR primer sets

For the calculation of the changes in relative copy number of the target genes under stress conditions, the 2^−ΔΔCT^ method was used^37^ where CT means the point at which the fluorescence signal crosses the threshold. ΔCT=CT (target gene)−CT (internal control: 16s rDNA was used as internal control in this study) and ΔΔCT=ΔCT Time *x*−ΔCT Time 0. For the ΔΔCT calculation method to be valid, it is important for the amplification efficiencies of the target and reference primer set to be approximately equal. Therefore, we initially verified that the primer sets to be used had approximately equal amplification efficiency to the Li primer set targeting Las 16s rDNA. Only those primer sets with approximately equal amplification efficiencies were chosen for further analysis.

### Data analysis

Based on the above primer amplification efficiency analysis, 2^−^^ΔΔCT^ method (Livak and Schmittgen, 2001) was used to determine the relative changes in copy number of the target genes under different stress treatments in this study. All qPCR Ct results displaying no detectable level of Las 16S rDNA were arbitrarily assigned the value of 40, indicating zero detection after 40 cycles (Las levels above Ct value = 36.9 using Taqman probe are considered negative^36^). The data presented are the mean values with standard error of three biological replicates. The effects of heat/tetracycline stress on both FP1 and FP2 copy number at each post-temperature shift (PTS) were first analyzed by analysis of variance (ANOVA), then pairwise comparison between treatments were determined by Tukey’s honestly significant difference tests. All statistical analyses were performed using the R V3.2.1 software (Team, 2015) with the level of significance set at 0.05.

## Results

### Heat stress eliminates HLB symptoms by increasing both FP1 and FP2 copy number

**Fig. 1:**
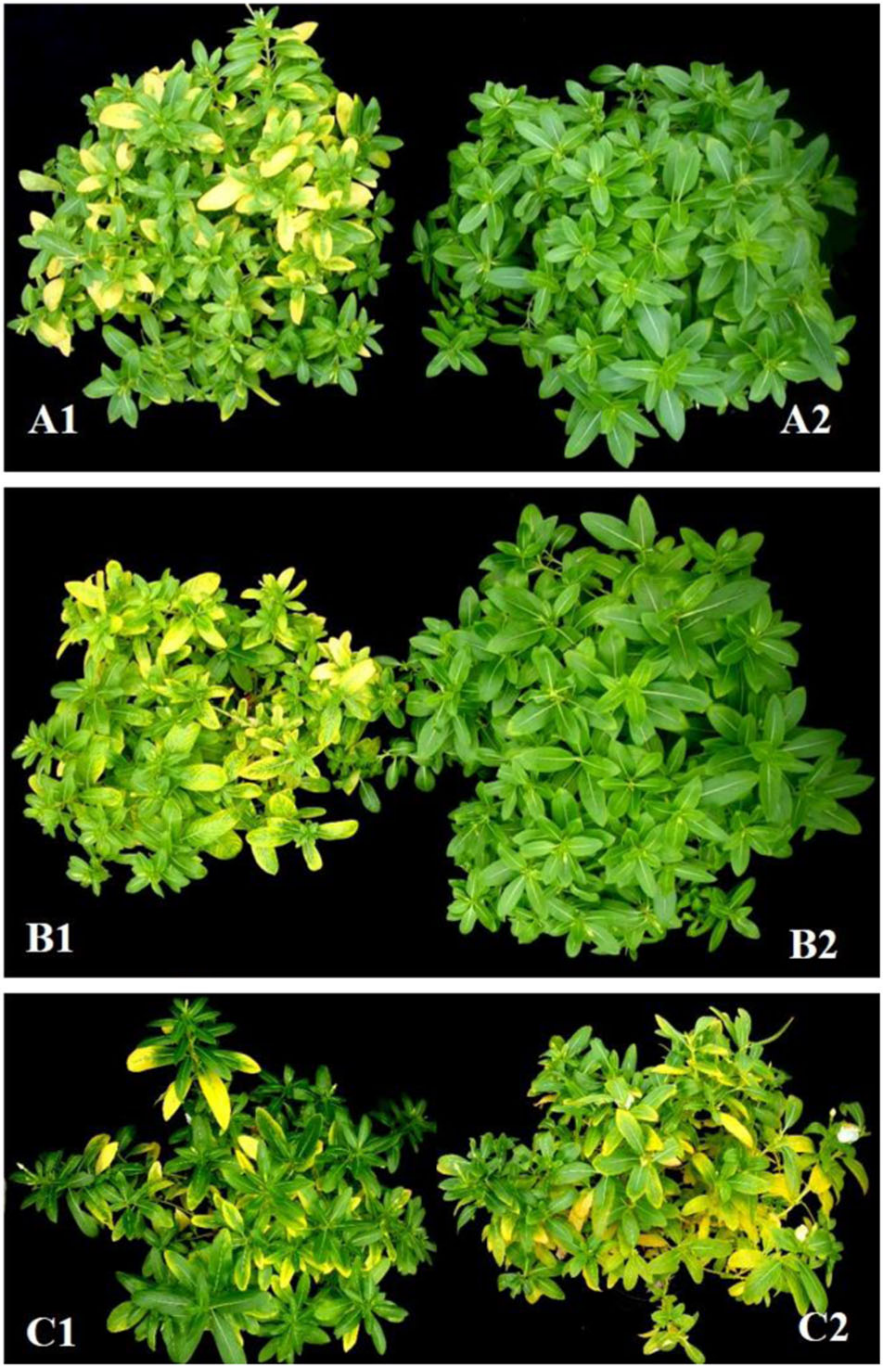
HLB-affected periwinkle plants before and after heat treatment. **a** Heat treatment at 42 °C for 10d (**a1**: before; **a2**: 3 months after); **b** heat treatment at 45 °C for 3 days (**b1**: before; **b2**: 3 months after). **c** No heat treatment control (**c1**: before; **c2**: 3 months after)

In order to investigate the mechanism of thermotherapy against Las, we first confirmed that the heat-treatment protocols used in this study resulted in the same outcome as the heat treatments from our previous study, despite slight temporal and temperature differences. Our previous study showed that heat treatment at 40 °C for 10 days was sufficient to reduce Las titer below detectable levels, as measured by real-time PCR with primers specific for Las 16S ribosomal DNA^18^. To verify that our new heat-treatment protocols eliminated HLB symptoms in a manner similar to the previous study, we subjected periwinkle (Fig. 1) and grapefruit (Figure S2) plants to heat treatments described above in Materials and methods. As expected, typical HLB symptoms of chlorosis and blotchy mottle were eliminated in heat-treated plants subjected to the newly developed 42° (Fig. 1a and S2A) or 45° (Fig. 1b and S2B) protocols, while untreated plants (Fig. 1c, Figure S2C) continued to show symptoms after 3 months. Consistent with the observed visual phenotypes, real-time PCR analysis showed that the Las 16S DNA was undetectable 3 months after heat treatment (data not shown). We concluded that the new heat-treatment protocols functioned similarly to eliminate Las infection and proceeded to investigate the underlying mechanisms of thermotherapy.

**Fig. 2:**
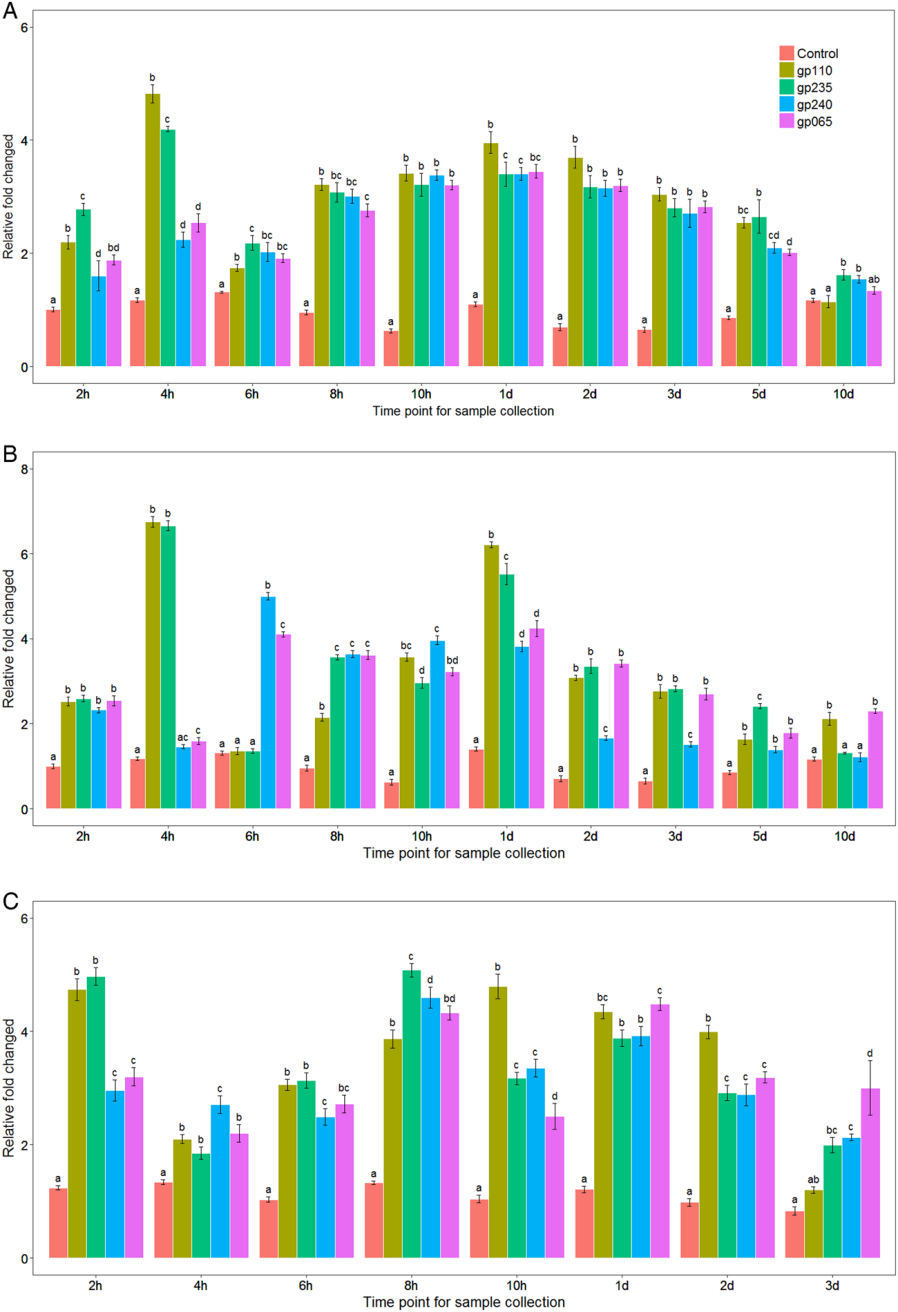
Relative copy number of FP1 and FP2 following different heat stress treatments. Total DNA was isolated from both periwinkle and citrus and subjected to real-time PCR analysis. The relative abundance of FP1-specific genes (gp-110 and gp-235) and FP2-specific genes (gp-240 and gp-065) was quantified and normalized to Las 16S ribosomal DNA after heat treatment at **a** 37 °C, **b** 42 °C, and **c** 45 °C. There is no significant difference between FP1 and FP2 prophages at the same temperature up shift treatment. But at different temperature up shift and different time point there is a significant difference among them (*P* < 0.05)

In order to determine whether sudden temperature increase could affect the release of FP1 and FP2 from lysogenic cells, we separately analyzed four FP1- and FP2-specific primer sets (gp_110, gp_235 for FP1 and gp_240, gp_065 for FP2) after three different temperature increase conditions: 37, 42, and 45 °C. A sudden shift from 23 to 37 °C caused the relative copy number of FP1 to increase by 2.49 ± 0.41-fold after only 2 h (Fig. 2a). Relative FP1 copy number reached its highest level at 4 h PTS, with 4.55 ± 0.38-fold induction. Relative FP2 copy number increased from 1.75-fold (±0.18; second hour PTS) to 2.39-fold (±0.18; fourth hour PTS), with the highest level of 3.42 ± 0.03-fold after 1 day at 37 °C. Both FP1 and FP2 showed an average increase of more than threefold at this time point. Interestingly, two phage induction peaks were observed for both FP1 and FP2 prophages (fourth hour and 1 day PTS; Fig. 2a). When exposed to 42 °C (Fig. 2b), relative FP1 abundance increased to 6.70 ± 0.70-fold at 4 h PTS and 5.87 ± 0.49 at 1 day PTS compared to the initial abundance. For FP2, relative abundance reached 4.54 ± 0.62-fold at 6 h PTS, with a secondary peak at 1 day PTS, 4.03 ± 0.30-fold that of the initial abundance. When shifted to 45 °C (Fig. 2c), the relative abundance of FP1 increased to 4.86 ± 0.4-fold of the initial abundance at 2 h PTS and reached a secondary peak at 8 h PTS, showing 4.48 ± 0.93-fold increase. The relative copy number of FP2 increased to 3.08 ± 0.41-fold the initial abundance at 2 h PTS and 4.47 ± 0.44-fold the initial abundance at 8 h PTS. When plants were continuously heated at 45 °C for 1 day, relative FP2 abundance was 4.2 ± 0.4-fold the initial value. In summary, exposure to 42 and 45 °C increased the relative abundance of FP1 and FP2 as compared to 37 °C, and there was a significant difference among three heat treatments when all the time points were analyzed by ANOVA (*P* < 0.05).

To confirm the above results, five primer sets targeting different prophage regions were also tested and their relative fold changes averaged. These were gp25 (putative phage tail fiber protein gene), gp30 (phage structural gene), gp35 (possible endolysin), gp200 (putative phage antirepressor gene), and rep-origin (putative phage replication origin, Pro R O). As a control, primers targeting the putative Las origin of replication were tested (Las R O). As seen in figure S3A (37 °C), figure S3B (42 °C), and figure S3C (45 °C), quantitation of these genes yielded similar results: two peaks in abundance at 4 h (4.13 ± 0.30) and 1 day (4.77 ± 0.51) after being shifted to 37 °C. After exposure to 42 °C, the abundance of both FP1 and FP2 increased from 2 h to 1 day PTS. Relative abundance of FP1 and FP2 peaked at 1 day PTS, with an average of 5.69 ± 0.77-fold increased abundance compared to the initial value. When shifted to 45 °C, a general pattern of increase was visible from 4 to 10 h PTS for the primer sets targeting prophage genes but not for the primers targeting Las replication origin (Las R O). During the three different heat stress treatments, relative FP1 abundance increased by an average of 3.21 ± 0.41-fold and FP2 abundance increased by 2.85 ± 0.98-fold. Unsurprisingly, the average relative abundance of the Las origin of replication compared to the Las 16S sequence consistently stayed near the expected value of 1.0. Interestingly, though, the average relative abundance of the phage origin of replication rose to several times its initial value, showing that heat stress can change the ratio of phage to Las. This altered ratio indicates that the dynamics of Las and prophage replication may be disturbed by entering into the lytic cycle.

### Both FP1 and FP2 actively respond to tetracycline stress

**Fig. 3:**
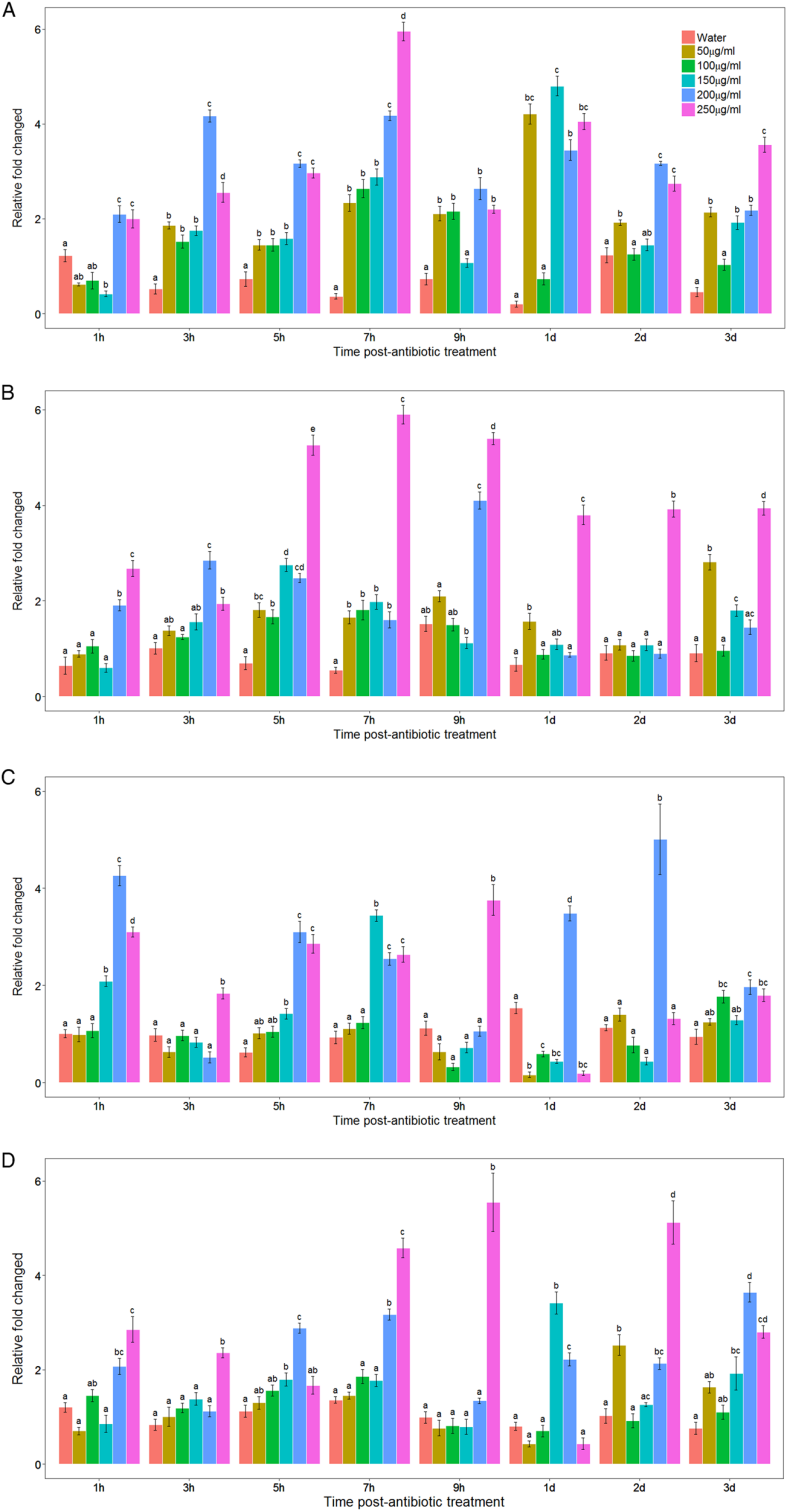
Relative copy number of FP1 and FP2 following tetracycline treatment. Total DNA was isolated from citrus leaves and subjected to real-time PCR analysis. The relative abundance of phage-specific genes was quantified and normalized to Las 16S ribosomal DNA after tetracycline treatment with 50, 100, 150, 200, or 250 µg/ml concentrations. **a** FP1-specific primers for gp-110 gene; **b** FP1-specific primers for gp_235 gene; **c** FP2-specific primers for gp_240 gene; **d** FP2-specific primers for gp-065 gene. Data are presented as mean ± S.E.M. (*n* = 3). There is no significant difference between FP1 and FP2 prophages at the same concentration of tetracycline treatment. But at different concentration of tetracycline and different time point, there is a significant difference between each other (*P* < 0.05)

To investigate the effects of antibiotic stress on the relative copy number of FP1 and FP2 in Las, several different concentrations of tetracycline were applied to plants by branch immersion. When lemon branches with typical HLB symptoms on their leaves were treated with 50–150 µg/ml (Fig. 3a, b) of tetracycline, maximum copy number for FP1 was observed at 1 day posttreatment, with relative copy number being 4.8 ± 0.87-fold the initial copy number (Fig. 3a, gp110 gene). The same treatment increased FP2 (Fig. 3c, d) relative copy number to the highest level of 3.4 ± 0.39-fold the initial value (Fig. 3c, gp240 gene) in 7 h. Increased tetracycline application at concentrations of 200 or 250 µg/ml yielded the greatest increases for FP1 and FP2 relative copy number, with maximum values of 5.95 ± 0.38-fold (Fig. 3a) and 5.55 ± 0.95-fold (Fig. 3d) after being treated for 7 and 9 h, respectively. Additionally, when treated by 200–250 µg/ml for 2 days, the relative copy number of FP2 reached another peak around fivefold (Fig. 3c, d). Relative copy number for structural genes of both phage FP1 and FP2 increased to a maximum of 8.92 ± 1.70-fold (Figure S4A, gp25 gene) and 7.26 ± 1.21-fold (Figure S4B, gp30 gene) after 7 h of treatment with 150 µg/ml tetracycline. This was consistent with the previous changes quantified by specific genes of FP1 and FP2 separately. For the endolysin gene (Figure S4C, gp35 gene) and the putative antirepressor (Figure S4D, gp200 gene), both reached the highest level when treated for 7 h with 150 µg/ml tetracycline, showing 5.80 ± 1.09 and 6.09 ± 1.37-fold changes, respectively, when compared to the initial values. In short, the relative phage copy number of both FP1 and FP2 was dramatically increased when exposed to tetracycline treatment. Untreated or water-treated control samples never showed variation of >1.7 ± 0.53-fold compared to the initial values. We therefore hypothesized that the altered ratio of phages to Las is caused by conversion from the lysogenic to the lytic pathway.

### TEM confirms temperature-dependent lysogenic to lytic conversion of prophage in Las

**Fig. 4:**
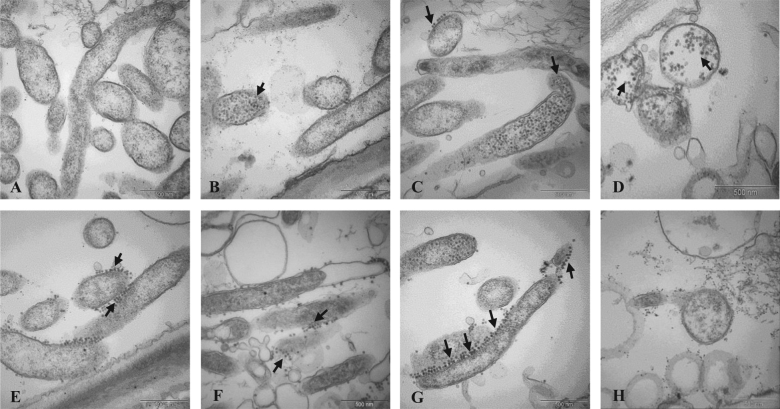
TEM images of Las and phage particle morphology after 45 °C heat stress treatment. **a** Control image of Las in periwinkle at 23 °C; phage particles are absent; **b**,** c** phage particles are visible 8 h after 45 °C temperature increase in Las-infected periwinkle; **d**,** e** phage particles are visible both inside and outside Las cells 3 days after 45 °C temperature increase; **f**,** g** phage particles released from Las cells 3 days after 45 °C temperature increase; **h** lysis of Las cells concurrent with release of phage particles 3 days after 45 °C temperature increase

Our qPCR data suggested that sudden temperature increase induced lysogenic to lytic conversion of prophages FP1 and FP2 in Las. Further evidence of lysogenic to lytic conversion was obtained by TEM. We observed the morphology and subcellular structures of Las, including phage induction during heat stress treatment. When exposed to 45 °C heat stress, lysogenic to lytic conversion was observed after 8 h, as evidenced by phage particles formed within Las cytoplasm (see arrow, Fig. 4b). The release of a small number of phage particles from the cell was also observed (Fig. 4c, see arrow). After 3 days of heat treatment, the number of phage particles released had increased greatly (Fig. 4d–g). In contrast, almost no phage particles were observed in unheated periwinkle samples used as control (Fig. 4a). Together with the qPCR data, these observations strongly support that phage induction is triggered by sudden temperature increase, which may contribute to the reduction of Las population during heat treatment.

## Discussion

A prophage, also known as a temperate phage, can integrate into the circular bacterial DNA chromosome, continuing this lysogenic cycle for as long as host physiology remains stable. However, stresses such as heat, UV light, starvation, or chemicals like antibiotics, which cause DNA damage to bacterial cells, activate the “SOS” stress response^38,39^ and cause some prophages to enter the lytic cycle, killing the bacterial host and releasing phage particles. In our previous study^18^ and the present study, we found that, when HLB-affected citrus or periwinkle plants were continuously exposed to 40 °C for 7 days or 45 °C for 3 days, HLB symptoms were eliminated and the titer of Las was greatly reduced or undetectable 3 months after heat treatment. Heat stress is known to cause the lytic induction of several bacteriophages, including one in *Xylella fastidiosa*, *Escherichia coli* phage-λ, and cyanophages of cyanobacteria^25,40–42^. Therefore, we hypothesized that environmental stresses may trigger the induction of prophage in Las, reducing the viability and number of the HLB-associated bacteria. To test this hypothesis, we subjected periwinkle plants to three different heat stress treatments. Each heat stress treatment resulted in a significant (*P* < 0.05) increase in the relative abundance of FP1 and FP2 compared to initial abundance, with higher temperatures resulting in greater increases.

Although the study of Las is currently hindered by the inability to grow the bacterium in a pure culture, Las is believed to be a relatively heat-sensitive bacterium^43,44^. Las belongs to the *Rhizobiaceae* family of α-proteobacteria and has similar genomic characteristics to *Rhizobium*^45,46^, which resides in soil and establishes inside root nodules, showing optimal growth at 25–28 °C^47^. If the optimal temperature for Las growth is similar to that of *Rhizobium* (25–28 °C), this may explain FP1's and FP2’s induction by elevated temperature. Our data suggest that both FP1 and FP2 are temperature-sensitive prophages. The relative copy number of FP1 and FP2 was higher at 42 °C than at 37 °C, but temperature increase to 45 °C did not increase copy number beyond that observed with 42 °C. This result is consistent with previous reports on the induction of λ-prophage, which is temperature-dependent and can be elevated by higher temperatures^48^. Interestingly, the induction of temperature-sensitive J7W-1 prophage/phage in *Bacillus thuringiensis* is significantly different between closely related host strains, suggesting that host gene products, not just phage gene products, may influence phage induction^49^. Future studies should clarify how Las phage dynamics are affected by host plants and Las strains.

It is well documented that the switch from lysogenic to lytic cycle is stimulated by the bacterial SOS stress response^50^, which is induced by specific antibiotics that interfere with cell wall synthesis^40,51^ and DNA replication^52^. Many antibiotics have already been shown to influence the induction of phages. For example, mitomycin C is well known for causing prophage induction, however, not all prophages respond to it^53^. Ampicillin is a beta-lactam antibiotic that was shown to enhance phage development and production^40,51,54^. Tetracycline is an antibiotic active against a wide range of both Gram-positive and -negative bacteria. Tetracycline was chosen for this study because it had already been established as effectively bacteriostatic against Las^55–60^. When tetracycline was used to treat HLB-affected budwood and absorbed at a concentration of 100 µg/ml for 2 h, or 50 µg/ml for 3 h, HLB leaf symptoms were greatly reduced^61,62^. Consistent with those findings, when we applied tetracycline at a concentration of 50 or 150 µg/ml for 7–9 h, the relative copy number of FP1 and FP2 greatly increased. Increasing the tetracycline concentration to 200 and 250 µg/ml caused higher relative copy number changes for both FP1 (Fig. 3b, gp235) and FP2 (Fig. 3d, gp065) at the same time point. For the structural genes of both phage FP1 and FP2 (gp25 and gp30), the relative copy numbers increased to the highest levels when treated with 150 µg/ml at the 7 h time point (Fig. 3a, b), in accordance with the changes in specific genes of FP1 and FP2, which were previously quantified separately.

Although lytic cycle phage particles were found in HLB-affected plant samples^30^, only Las prophage FP1 encodes putative lysis genes (gp_110, gp_035). Yet our data showed that FP2 copy number increased similarly to FP1, despite the lack of putative lysis genes in FP2. The Las chromosome itself encodes a lytic enzyme (CLIBASIA_04790); further study should determine whether excision of FP1 and FP2 relies on the Las chromosomal endolysin and/or the FP1 putative endolysin. Slight temporal differences between FP1 and FP2 induction, as observed with 42 °C temperature shift (e.g., Fig. 2b), suggest that there may be small differences in their regulation. Future studies should identify the mechanisms underlying these differences.

Another direction for future study relates to the sometimes disparate results obtained with the two primer sets for FP1 (gp110, gp235) or FP2 (gp240, gp065). We used two primer sets for each phage because Las prophage regions are known to be highly variable (data not shown), and owing to this variability, it is unclear which gene’s copy number most accurately reflects the true phage copy number. Further studies may clarify how the copy number of specific phage genes correlates with the overall number of phage particles.

In conclusion, both heat treatment and tetracycline can induce FP1 and FP2 conversion. Although heat treatment and tetracycline have been demonstrated to be effective against HLB, this is the first study to suggest a possible molecular mechanism, in addition to citrus host transcriptional or metabolic defensive responses to stress conditions. All together may contribute to the elimination of Las. Our results suggest a potential mechanism for the activity of heat treatment and antibiotics against HLB, wherein induction of Las prophages causes lysis of Las bacteria, reducing Las population and thus HLB symptoms in citrus trees.

## Electronic supplementary material

Relative copy number of phage structural and putative functional genes following tetracycline treatment

Figure S3A

Figure S3B

Figure S3C

Figure S4A

Figure S4B

Figure S4C

Figure S4D
