# Supplementary material for: Molecular mechanisms underlying heat or tetracycline treatments for citrus HLB control
Source: Hortic Res. 2018 Jun 1;5:30. doi: 10.1038/s41438-018-0038-x (PMC5981314; doi:10.1038/s41438-018-0038-x)
Supplement: Supplementary file 1 — Relative copy number of phage structural and putative functional genes following tetracycline treatment [file 41438_2018_38_MOESM1_ESM.docx]

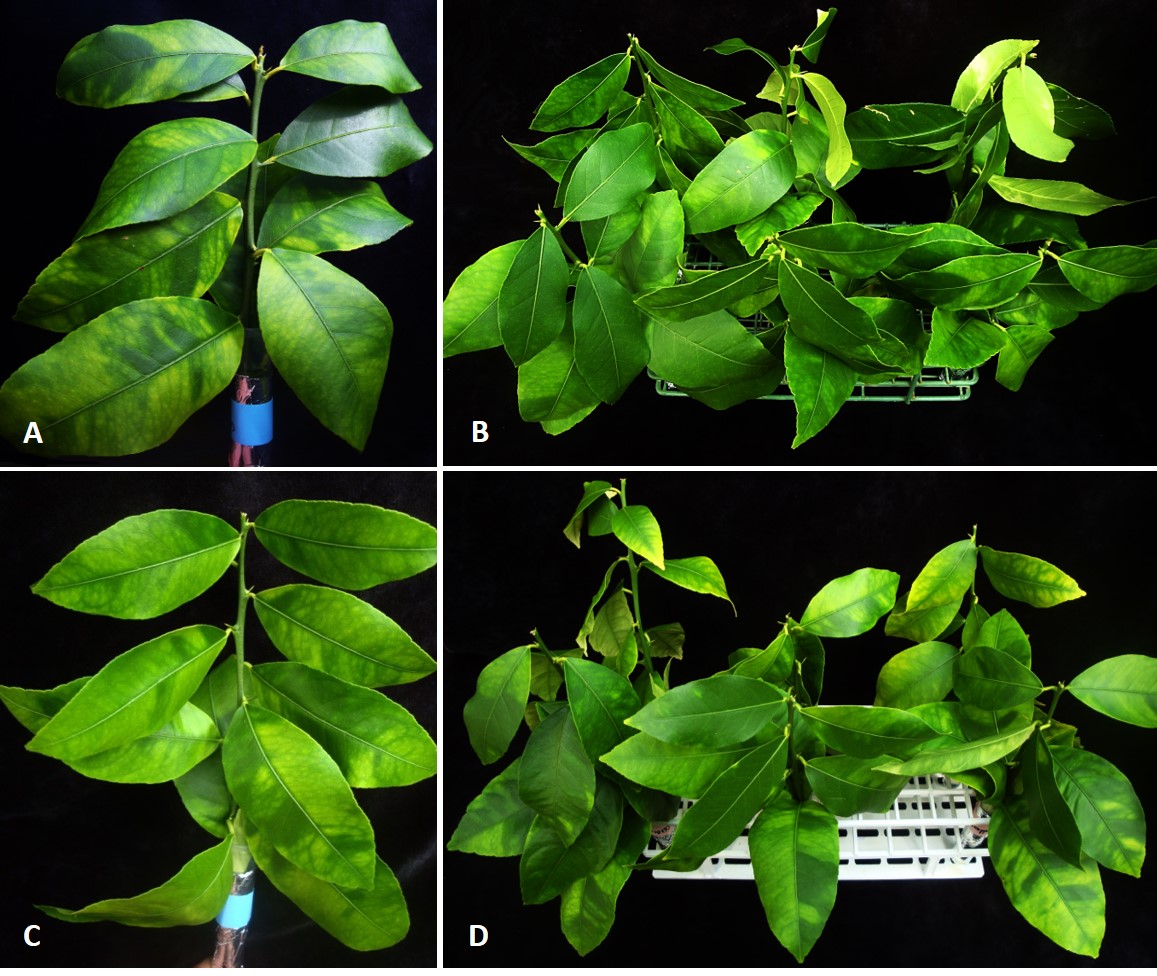


**Fig.S1.** **Lemon (*Citrus limon*) branches with typical blotchy mottle symptom used in this study for tetracycline treatment.** **A** and **C** show the individual branches dipped into tubes contain tetracycline covered with aluminum foil paper. **B** and **D** show lemon branches treated by different concentrations of tetracycline.


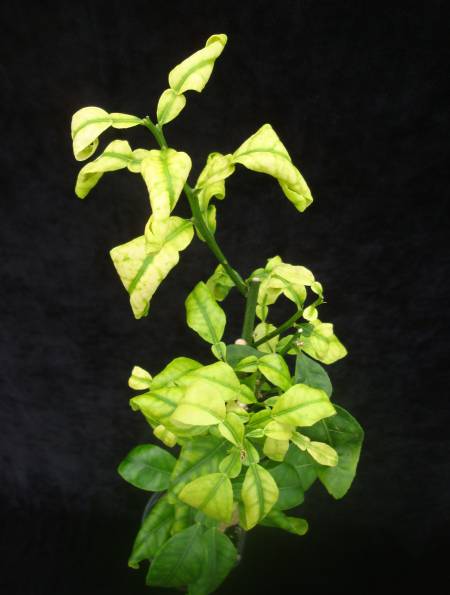

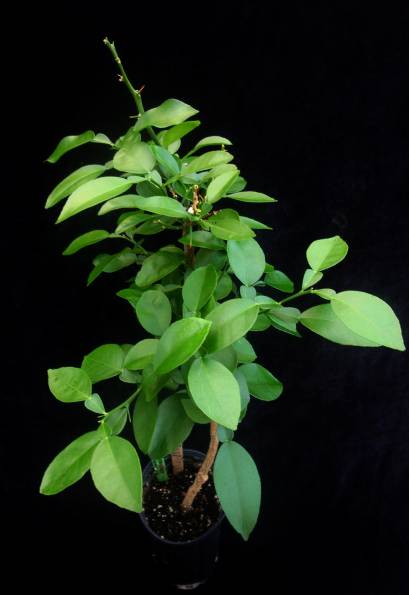

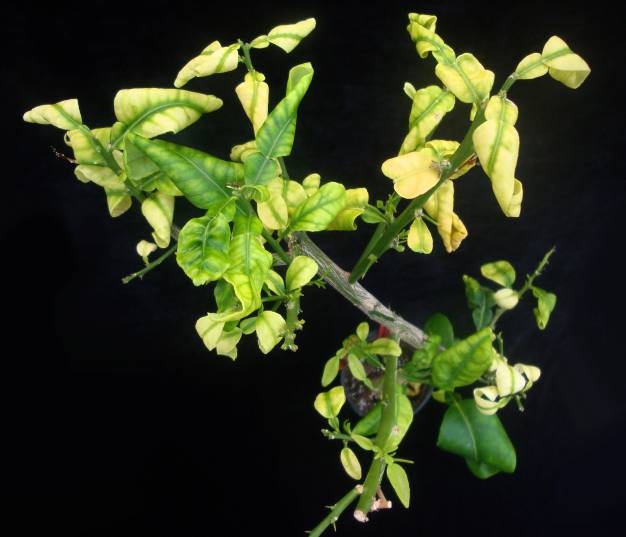

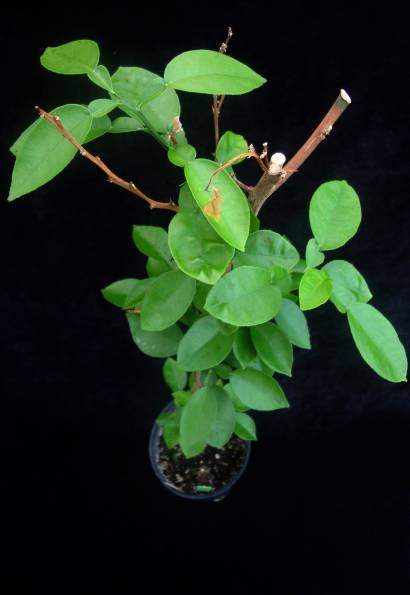

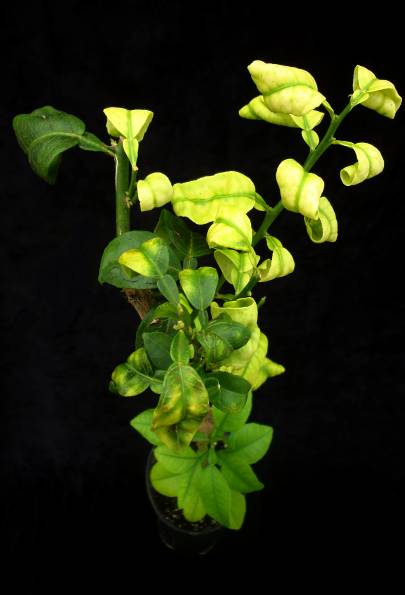

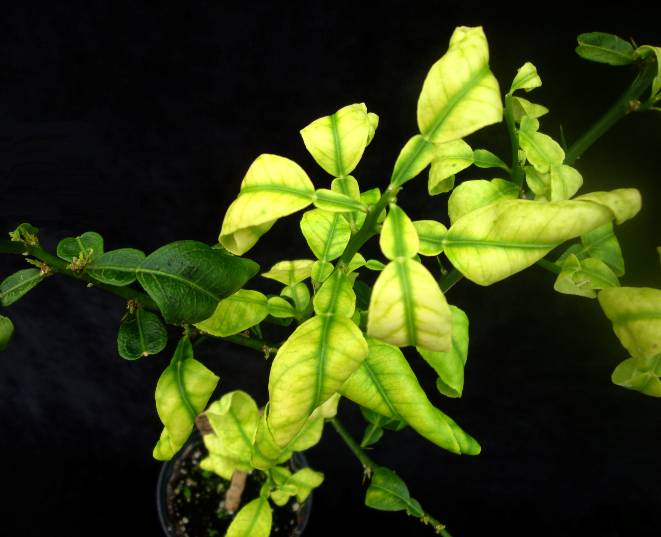


**A1**

**A2**

**B1**

**B2**

**C1**

**C2**

**Figure S2.** **HLB-affected grapefruit (*Citrus paradisi*) samples before and after heat treatment.** **A**, Heat treatment at 42°C for 10d (A1: before; A2: 3M after); **B**, Heat treatment at 45°C for 3d (B1: before; B2: 3M after). **C**, No heat treatment control (C1: before; C2: 3M after).

**S3A.**
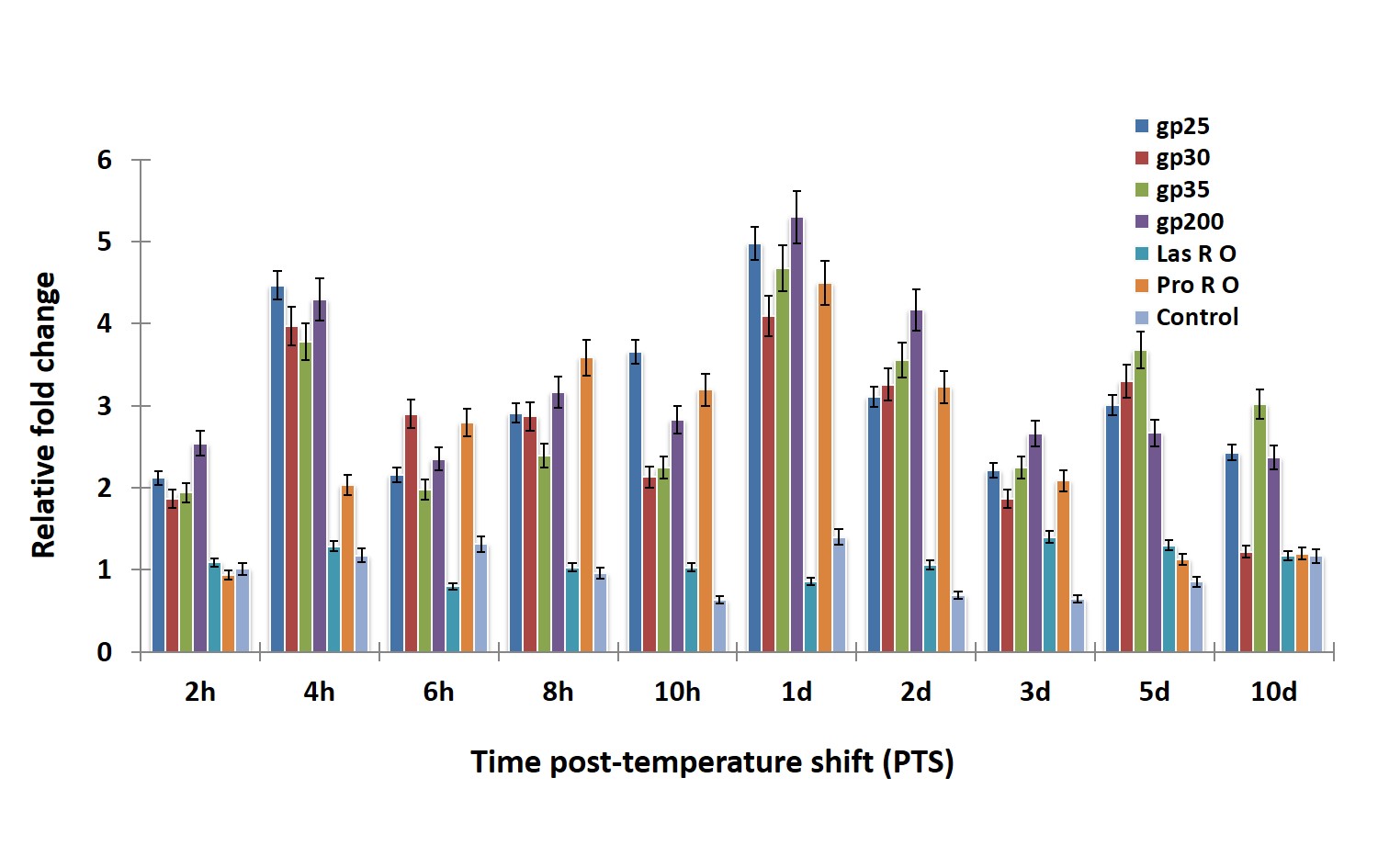


**S3B.**


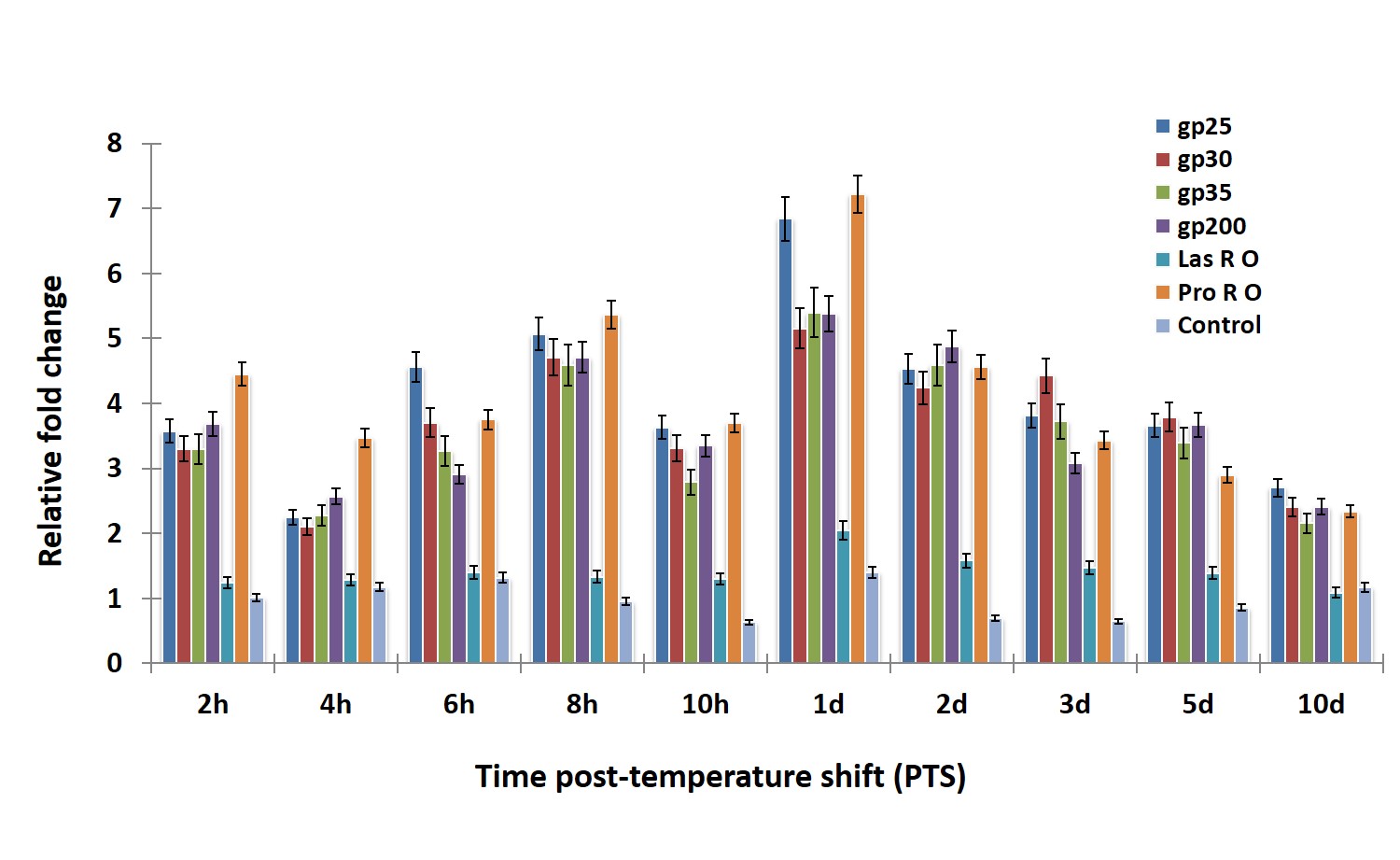


**S3C.**
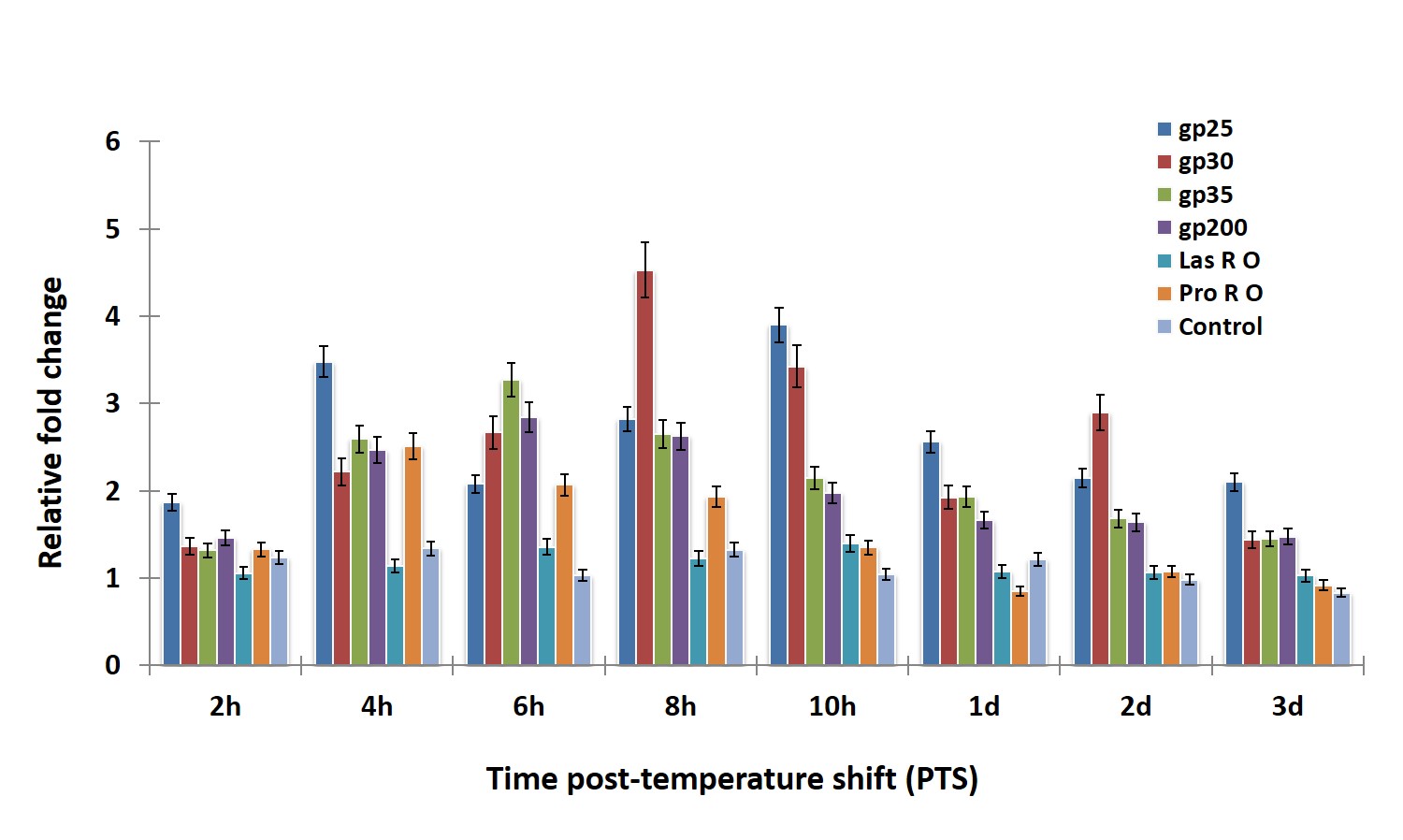


**Figure S3.** **Relative copy number of phage structural and putative functional genes following different stress heat treatment.** Relative abundance of FP1 and FP2 phage structural genes (gp-25 and gp-30), endolysin gene (gp-35), putative antirepressor gene (gp-200), Las origin of replication (Las R O), and prophage origin of replication (Pro R O) were quantified in the same manner after heat treatment at **A**, 37°C, **B**, 42°C, and **C**, 45°C. Control bars represent the average fold-change of all genes for untreated trees at 23°. Data is presented as mean ± S.E.M. (n=3).

**S4A.
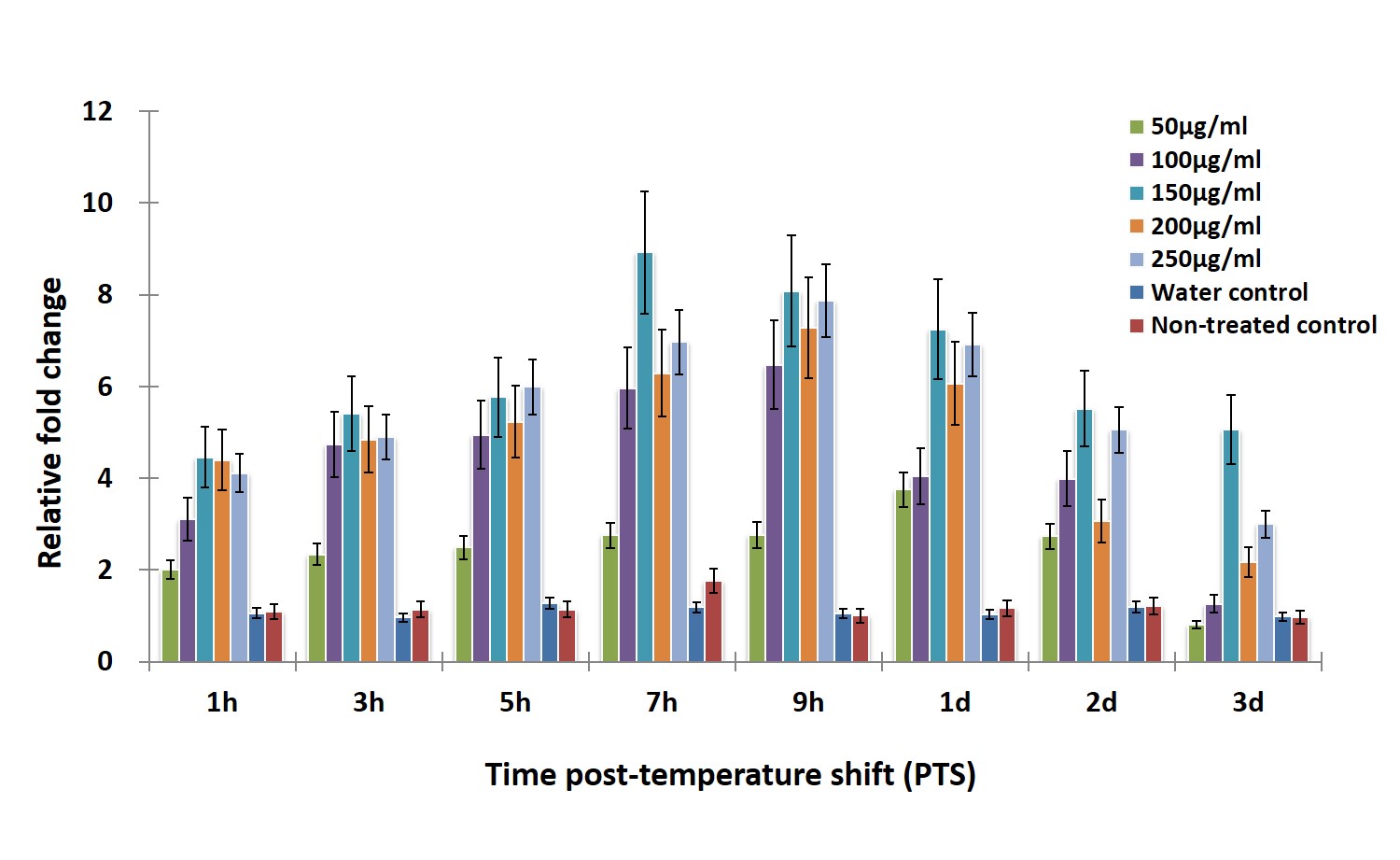
**

**S4B.**

**
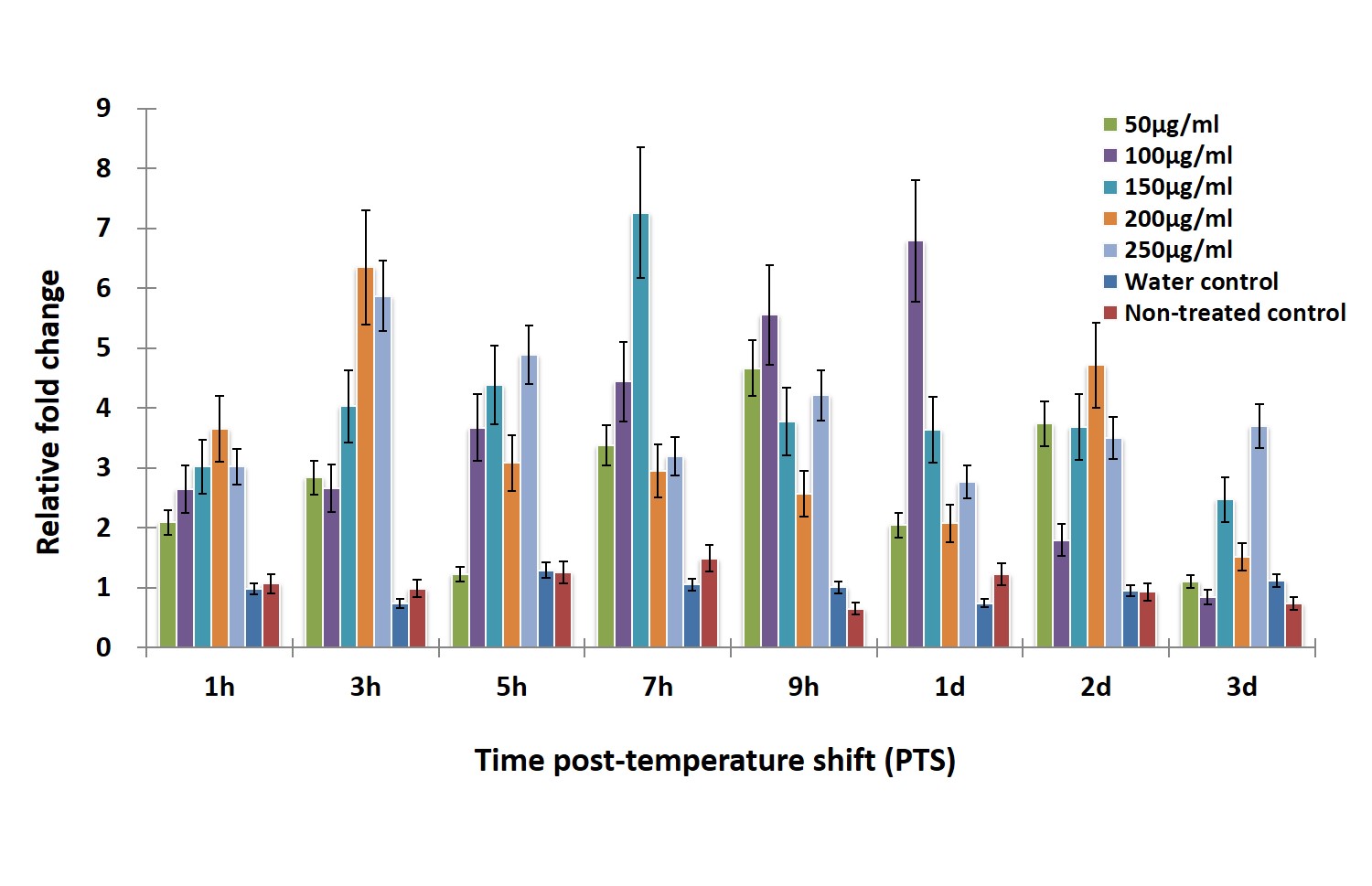
**

**S4C.
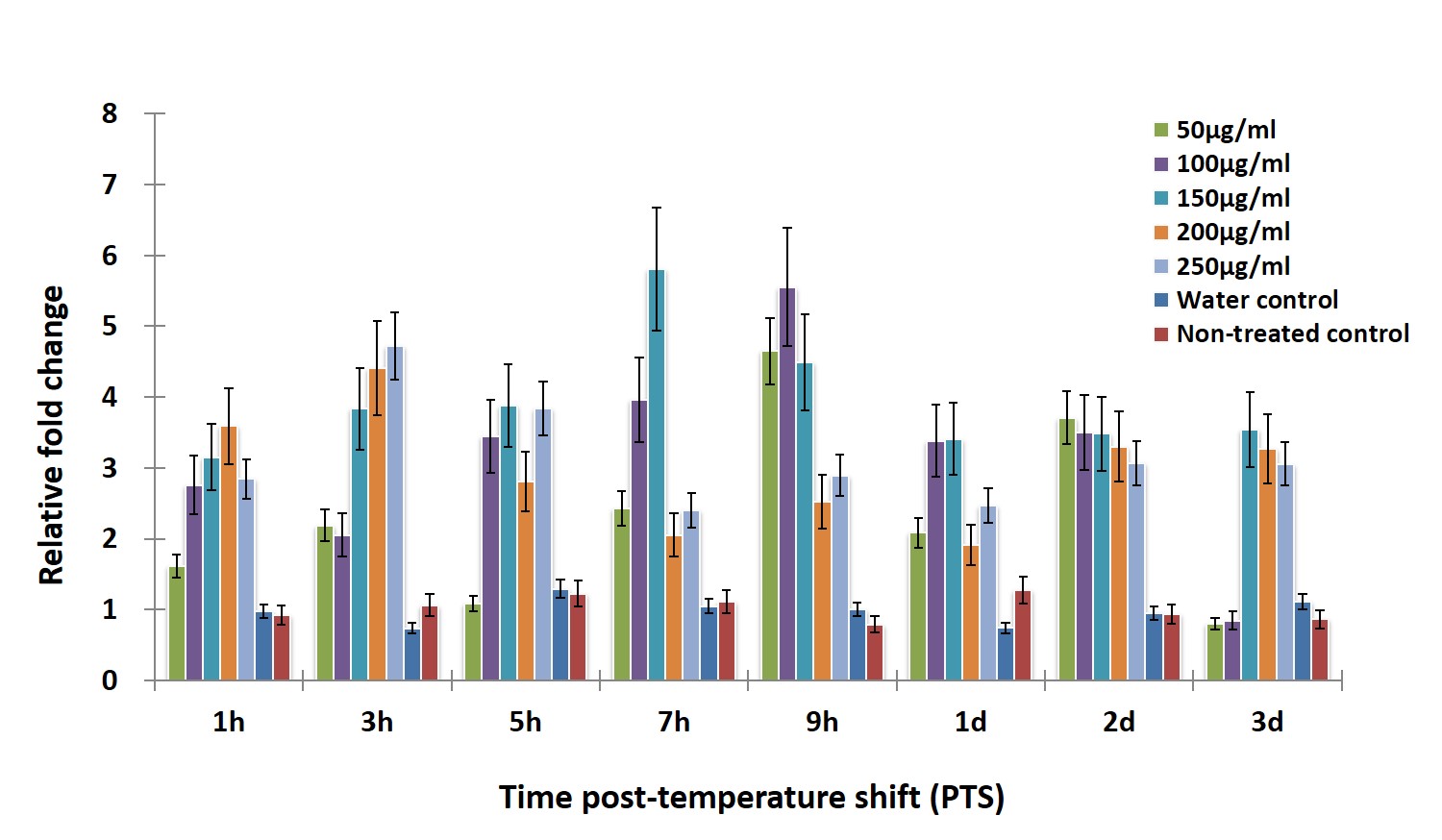
**

**S4D.**

**
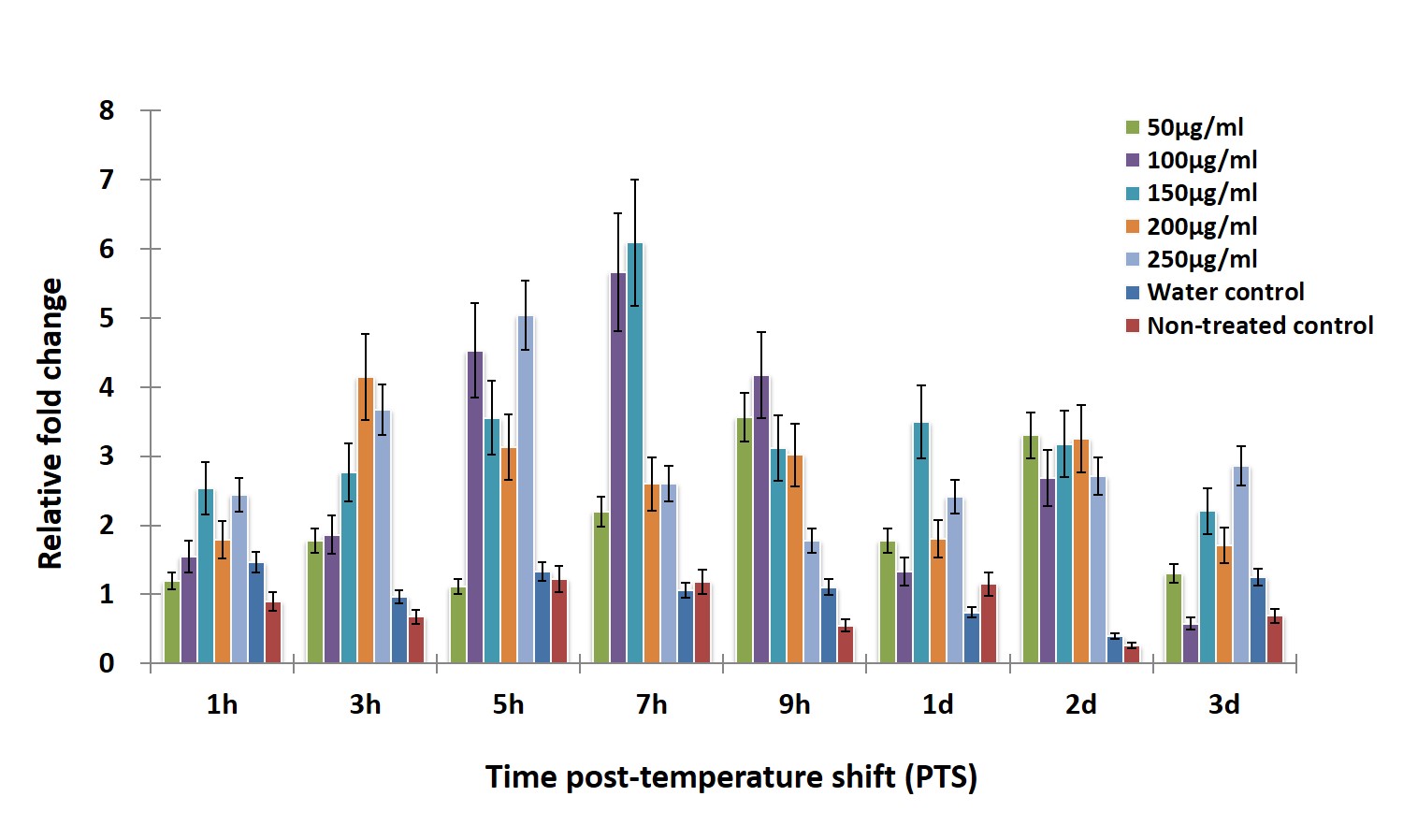
**

**S4E.
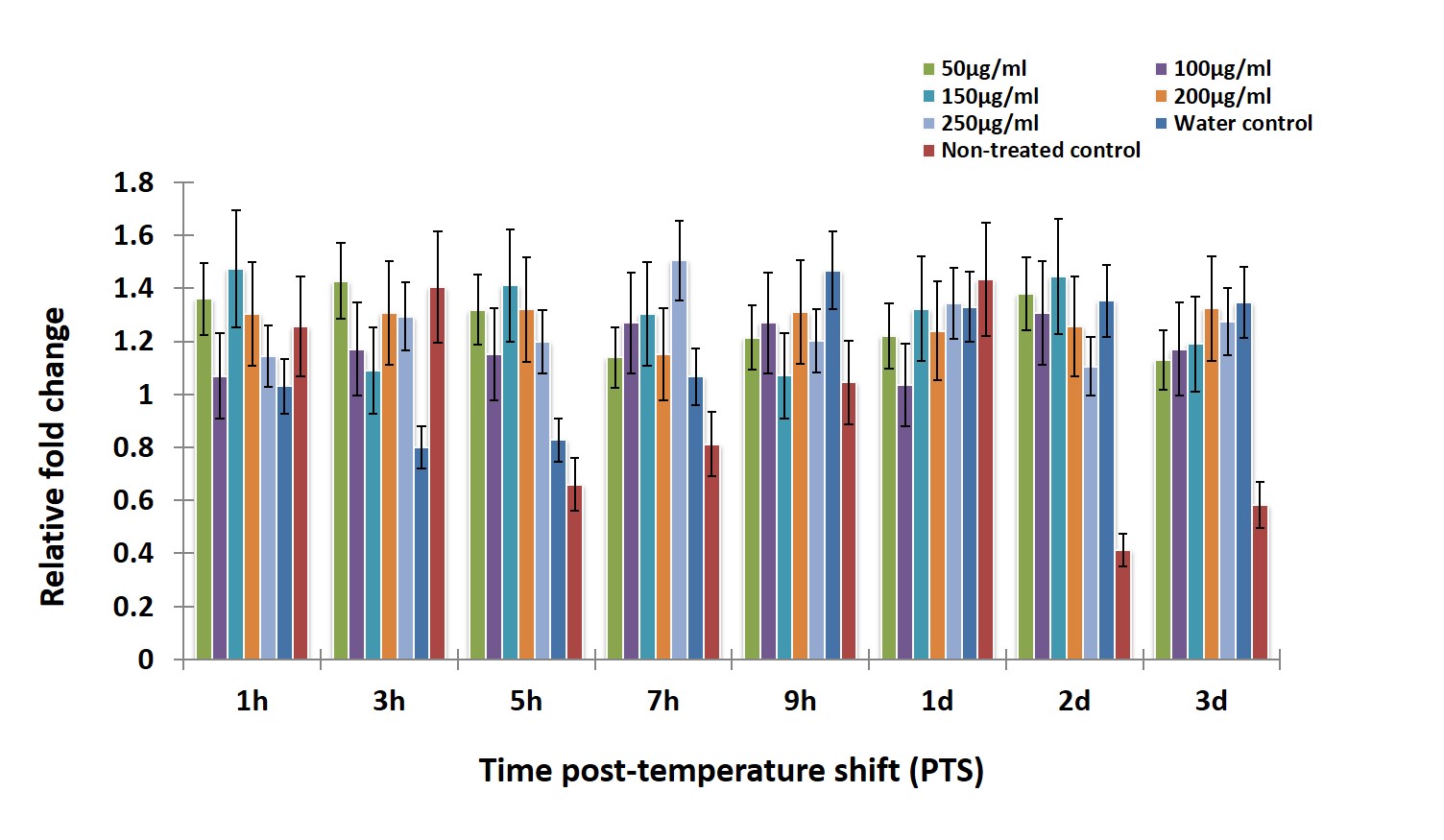
**

**S4F.**

**
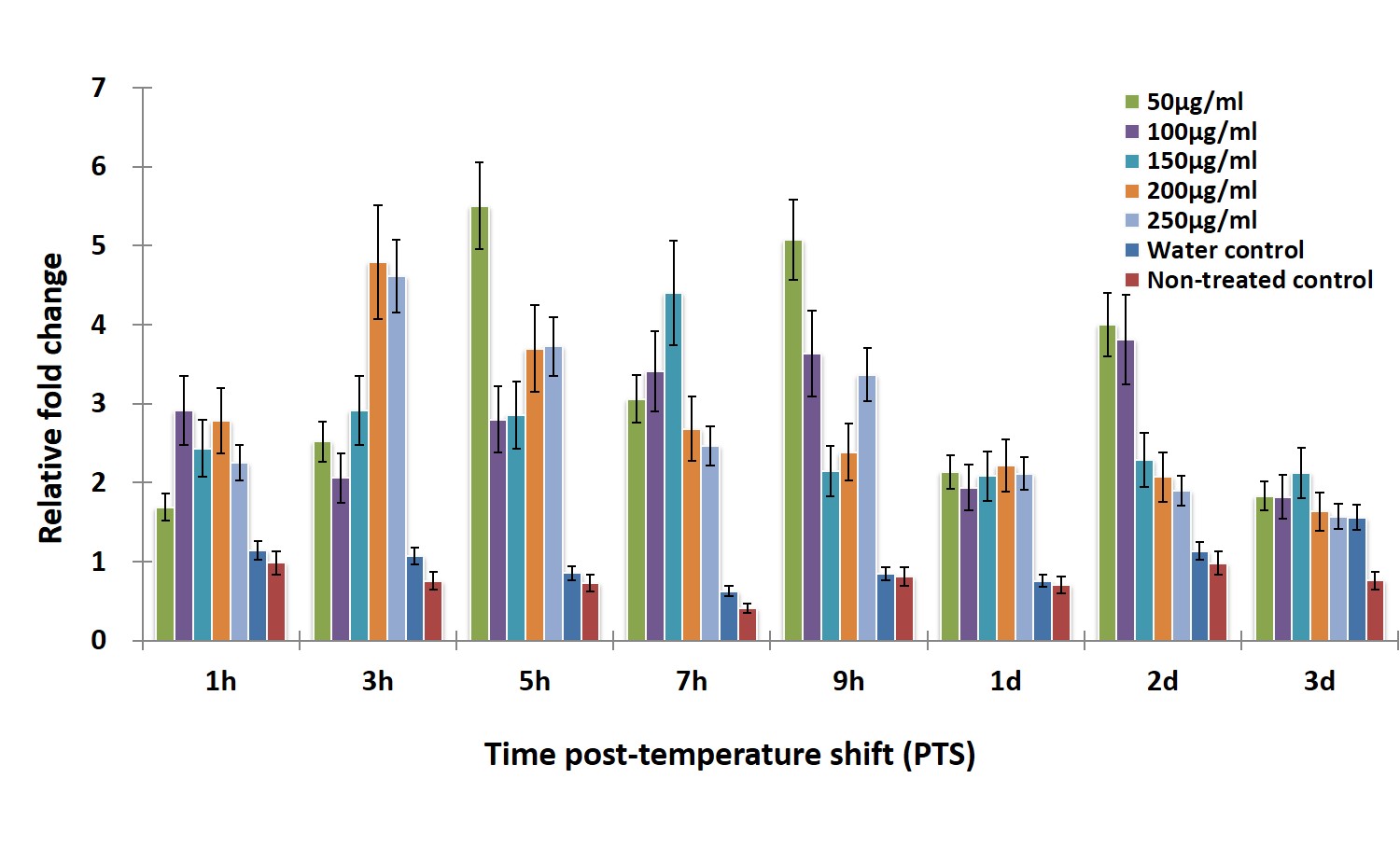
**

**Figure S4. Relative copy number of phage structural and putative functional genes following tetracycline treatment.** Total DNA was isolated from plant tissues and subjected to real time PCR analysis. The relative abundance of phage-specific or Las-specific genes was quantified and normalized to Las 16S ribosomal DNA after tetracycline treatment with 50, 100, 150, 200, or 250 µg/mL concentrations. **A**, FP1 and FP2 structural gene gp-25; **B**, FP1 and FP2 structural gene gp-30; **C**, putative endolysin gene gp-35; **D**, putative antirepressor gene gp-200; **E**, Las origin of replication; **F,** phage origin of replication. Data is presented as mean ± S.E.M. (n=3).

D

C
