# Supplementary figures and images for: Molecular mechanisms underlying heat or tetracycline treatments for citrus HLB control
Source: Hortic Res. 2018 Jun 1;5:30. doi: 10.1038/s41438-018-0038-x (PMC5981314; doi:10.1038/s41438-018-0038-x)

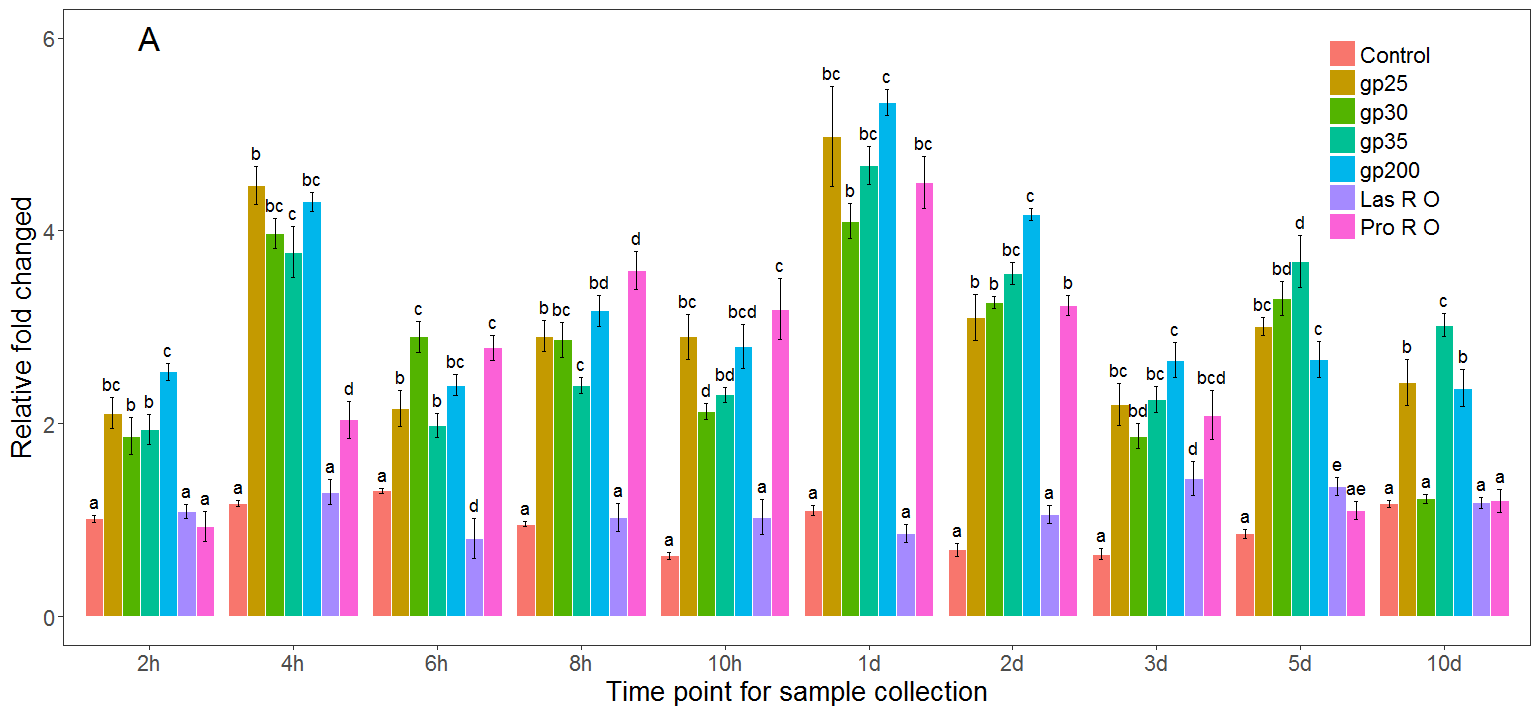

Supplement: Supplementary file 2 — Figure S3A [file 41438_2018_38_MOESM2_ESM.tif]

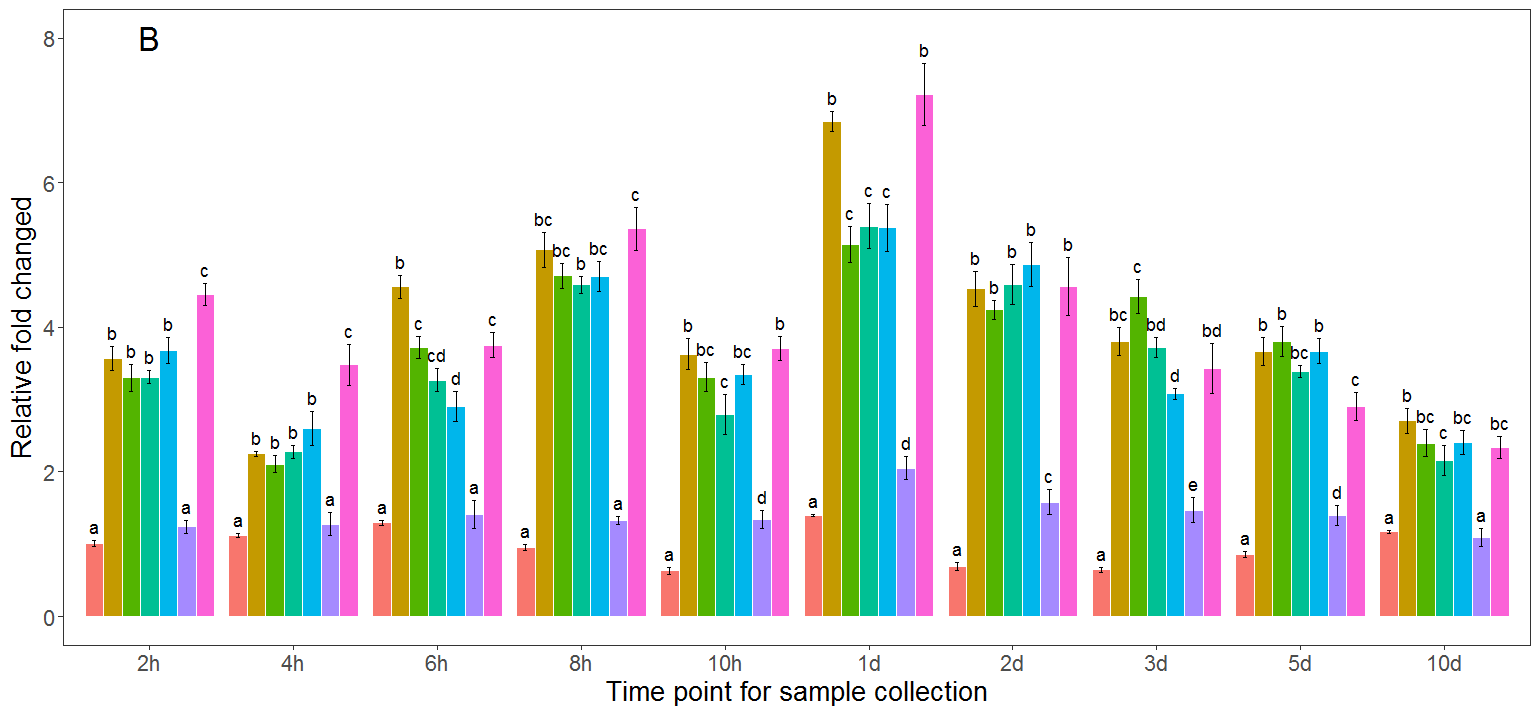

Supplement: Supplementary file 3 — Figure S3B [file 41438_2018_38_MOESM3_ESM.tif]

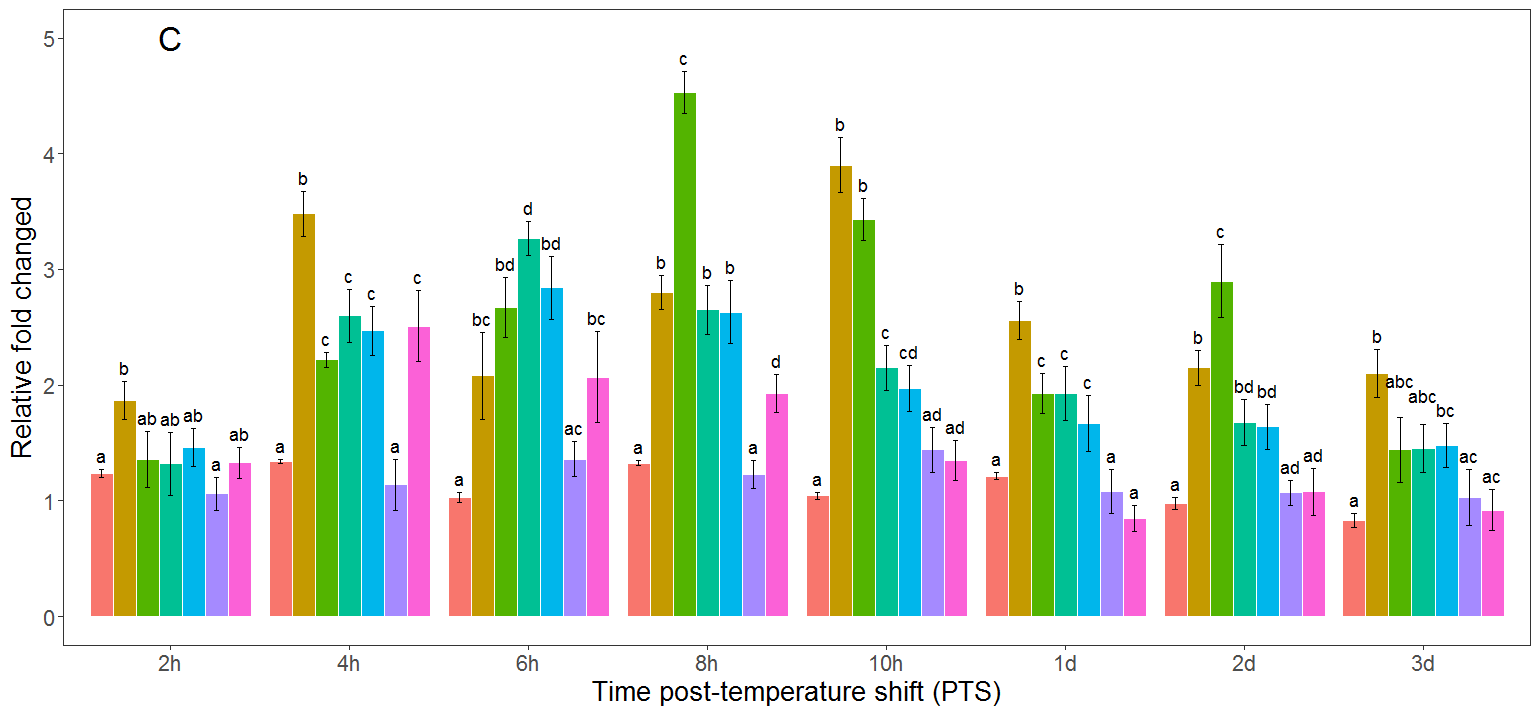

Supplement: Supplementary file 4 — Figure S3C [file 41438_2018_38_MOESM4_ESM.tif]

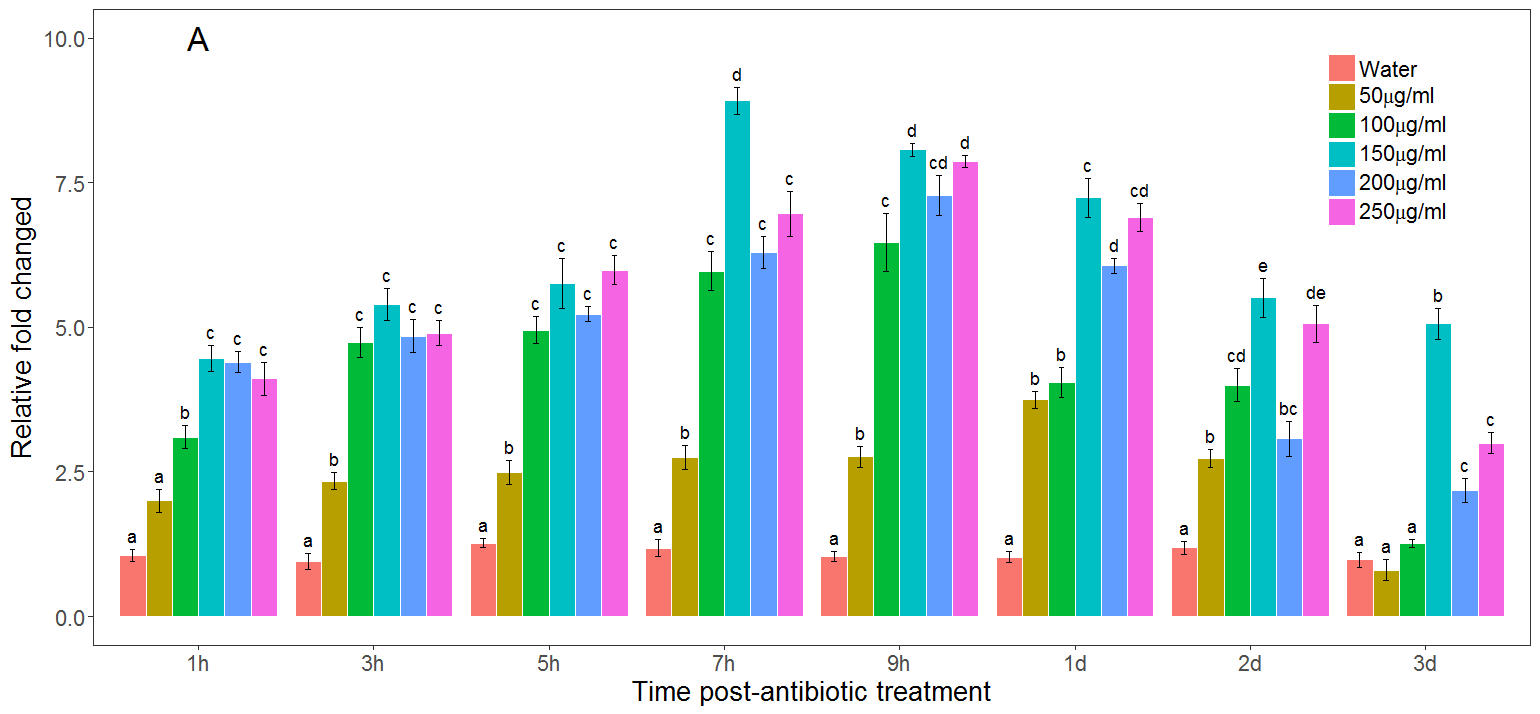

Supplement: Supplementary file 5 — Figure S4A [file 41438_2018_38_MOESM5_ESM.tif]

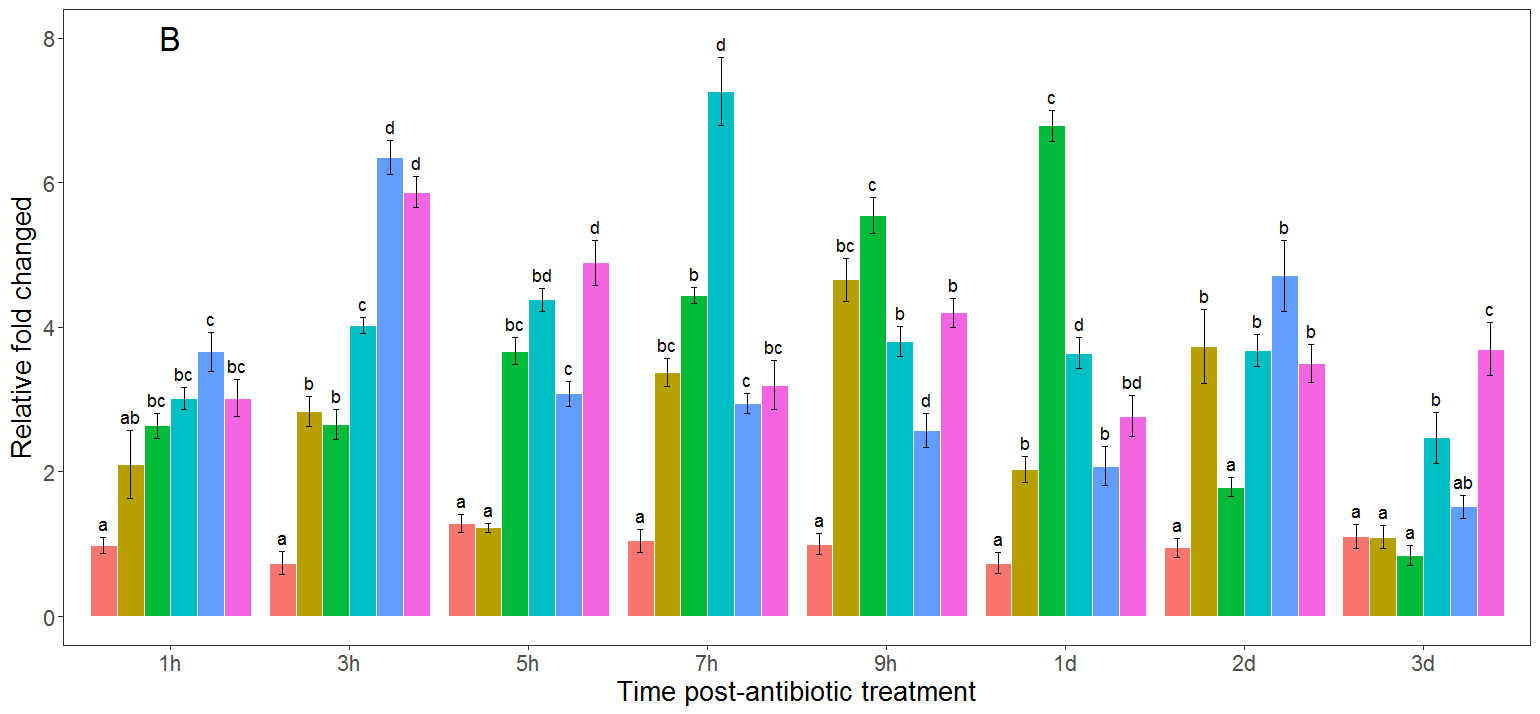

Supplement: Supplementary file 6 — Figure S4B [file 41438_2018_38_MOESM6_ESM.tif]

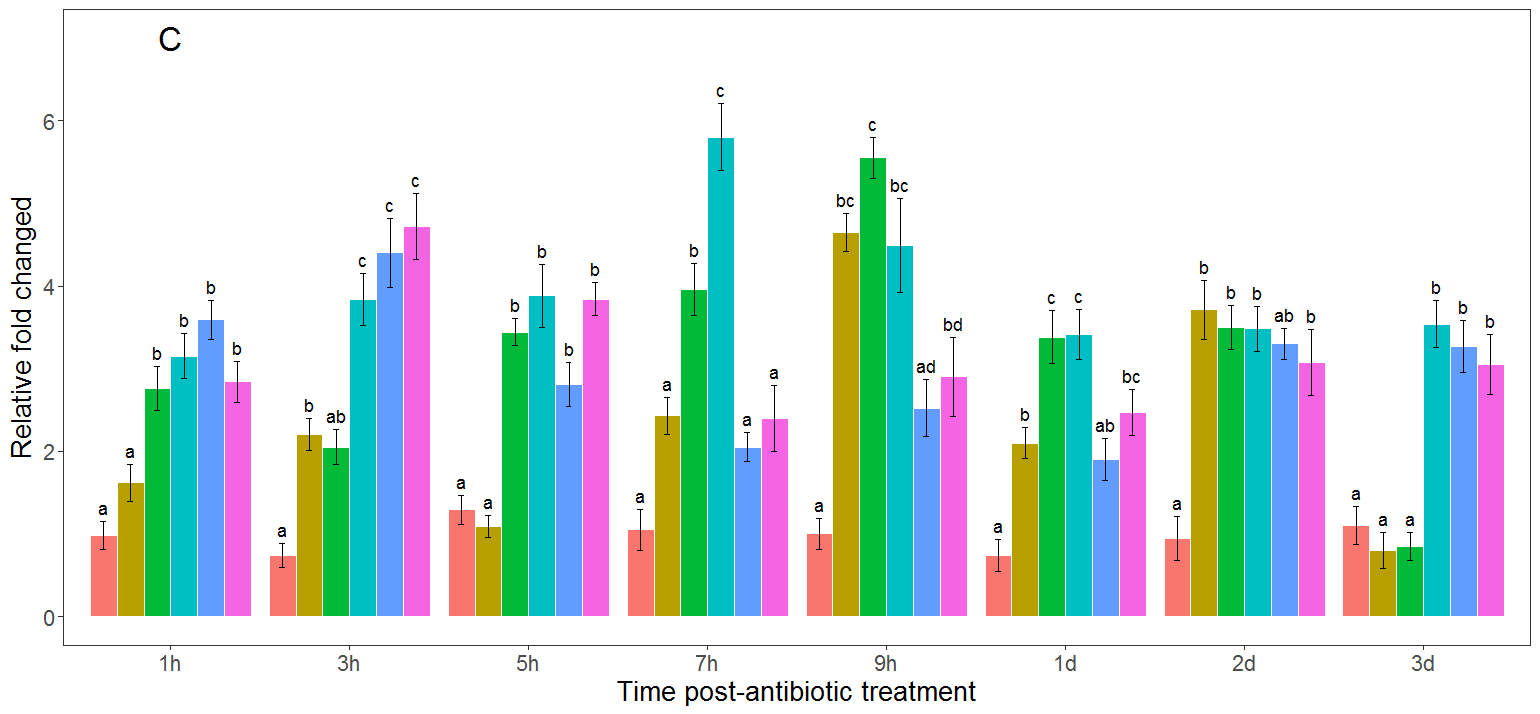

Supplement: Supplementary file 7 — Figure S4C [file 41438_2018_38_MOESM7_ESM.tif]

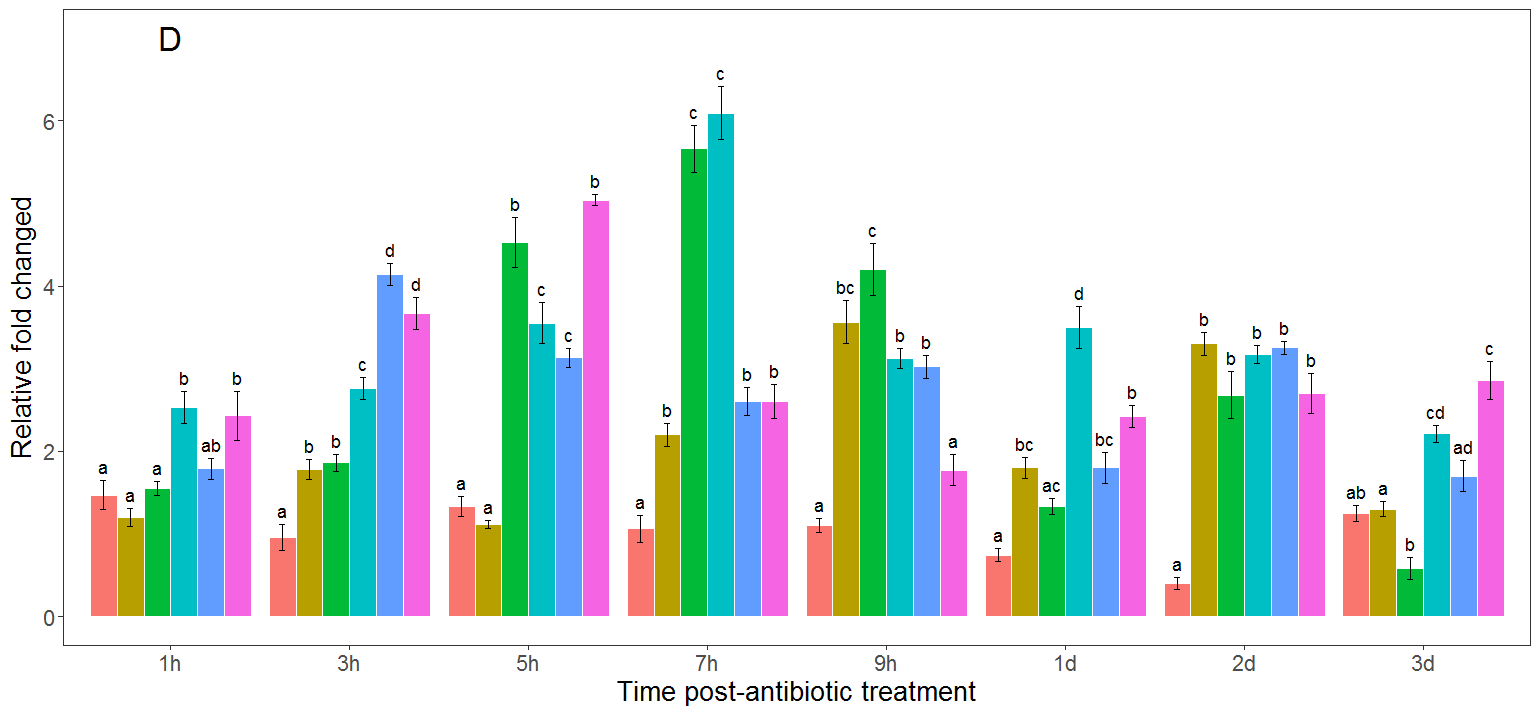

Supplement: Supplementary file 8 — Figure S4D [file 41438_2018_38_MOESM8_ESM.tif]
